# Marine Organisms as a Prolific Source of Bioactive Depsipeptides

**DOI:** 10.3390/md21020120

**Published:** 2023-02-11

**Authors:** Mingyuan Zeng, Jianyun Tao, Shuang Xu, Xuelian Bai, Huawei Zhang

**Affiliations:** 1 School of Pharmaceutical Sciences, Zhejiang University of Technology, Hangzhou 310014, China; 2 College of Life and Environmental Sciences, Hangzhou Normal University, Hangzhou 311121, China

**Keywords:** depsipeptide, marine cyanobacterium, sponge, mollusk, marine microbe, alga, biological activity

## Abstract

Depsipeptides, an important group of polypeptides containing residues of hydroxy acids and amino acids linked together by amide and ester bonds, have potential applications in agriculture and medicine. A growing body of evidence demonstrates that marine organisms are prolific sources of depsipeptides, such as marine cyanobacteria, sponges, mollusks, microorganisms and algae. However, these substances have not yet been comprehensively summarized. In order to enrich our knowledge about marine depsipeptides, their biological sources and structural features, as well as bioactivities, are highlighted in this review after an extensive literature search and data analysis.

## 1. Introduction

Marine organisms are tremendously important sources of natural products since almost 40,000 compounds have been discovered and recorded in the MarinLit database (https://marinlit.rsc.org/, accessed on 12 December 2022) [1]. Depsipeptides are an important group of polypeptides simultaneously containing ester and amide bonds, and they display a wide variety of biological properties [2,3]. A number of naturally occurring depsipeptides have been successfully developed as new drugs or are being evaluated in clinical trials, such as the antitumor agents romidepsin [4,5], plitidepsin (aplidine) [6,7], kahalalide F [8,9] and OBP-801 (spiruchostatin A) [10]. Generally, these substances are divided into two groups, namely, cyclic and non-cyclic, of which the former tends to display excellent bioactivity [11,12]. However, marine-derived depsipeptides have not yet been comprehensively summarized until now. In order to enrich our knowledge about these compounds, their origins and structural features, as well as their biological properties, are highlighted in this review.

**Figure 1:**
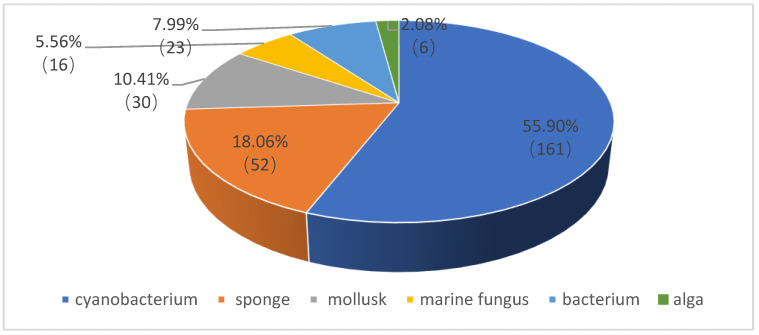
Percentage distribution of depsipeptides from marine organisms.

According to an extensive literature search using the DNP (Dictionary of Natural Products) database as well as Web of Science and SciFinder tools, as many as 288 depsipeptides (**1**–**288**) have been isolated and characterized from marine organisms. As shown in Figure 1, the major producers of depsipeptides are marine cyanobacteria, which make up 55.90%, followed by marine sponges (18.06%), mollusks (10.41%), bacteria (7.99%), marine fungi (5.56%) and algae (2.08%). On the basis of biological sources and chemical structures, these marine depsipeptides are each introduced herein. Their detailed information is supplied in the Supplementary Materials.

## 2. Marine Cyanobacteria

Marine-cyanobacterium-derived depsipeptides (**1**–**161**) have diverse chemical structures and a wide variety of pharmacological activities, and most of them are cytotoxic [13]. Structurally, these metabolites are linear and cyclic depsipeptides containing ɑ-amino or ɑ-hydroxy carboxylic acid residues, and the latter are the major components and can be further divided into five subgroups, including cyclic penta-, hexa-, and hepta-depsipeptides, thiazole-containing depsipeptides and others.

### 2.1. Linear Depsipeptides

**Figure 2:**
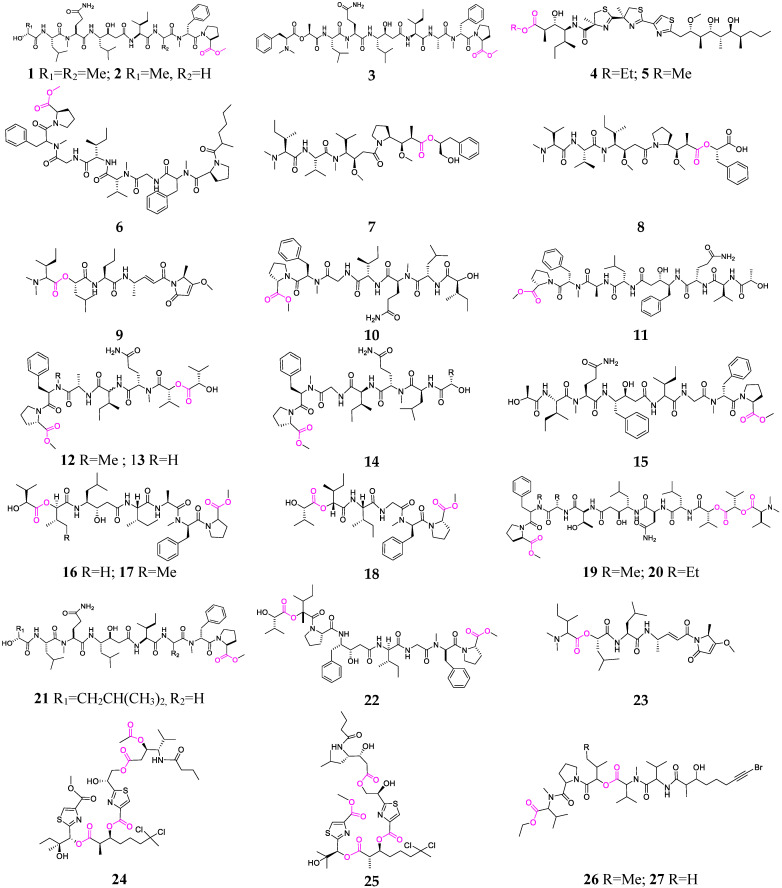
Marine-cyanobacterium-derived linear lipopeptides (**1**–**27**).

The marine cyanobacteria *Symploca* and *Lyngbya* spp. are the major producers of linear depsipeptides (**1**–**27**, Figure 2) [10,11,12,13,14,15,16,17,18,19,20,21,22,23,24,25]. Grassystatins D–F (**1–3**) containing statine units have strong aspartic protease inhibitory activity preferentially targeting cathepsins D and E [14]. Both compounds **4** and **5** possess an acetate extended and S-adenosyl methionine-modified isoleucine unit, a central triheterocyclic system comprising two *R*-methylated thiazolines and one thiazole, and a highly oxygenated and methylated C-15 polyketide unit [15], and **5** exerted potent inhibitory activity against the p53/MDM2 interaction (EC_50_ = 4.5 μM), an attractive target for anticancer drug development [15,16]. Malevamide D (**7**) exhibited a highly cytotoxic effect on P-388, A-549, HT-29 and MEL-28 cell lines in the subnanomolar range [17]. Symplostatins 3 (**8**) and 4 (**9**) were discovered as new analogs of dolastatin 10 and were shown to possess excellent cytotoxicity mediated by the disruption of microtubules [18]. Tasiamides A–E (**10**–**14**), produced by a *Symploca* sp., were found to have strong cytotoxicity against KB and LoVo cells [19,20,21].

The key structural feature of tasiamide F (1**5**) is the presence of a Phe-derived statine core, which contributes to its aspartic protease inhibitory activity [22]. Izenamides A, B and C (**16**–**18**) were purified from an Okinawan *Lyngbya* sp. and demonstrated an inhibitory effect on cathepsin D [23]. Grassystatins A–C (**19**–**21**) showed potency and selectivity against cathepsins D and E in vivo [24]. Maedamide (**22**) was reported as a novel chymotrypsin-inhibiting depsipeptide and strongly inhibited the growth of HeLa and HL60 cell lines [25]. Lyngbyabellins D (**23**) and P (**24**) are, respectively, produced by *Lyngbya* sp. and *Okeania* sp. and displayed strong antifouling and cytotoxic activities [26,27]. Gallinamide A (**25**) was presented as a new antimalarial pentapeptide from a *Schizothrix* sp. collected off the north coast of Panama [28]. Veraguamides K (**26**) and L (**27**) are two unique cytotoxic depsipeptides containing brominated alkynyls and were isolated from *Oscillatoria margaritifera* [29].

### 2.2. Cyclic Depsipeptides

#### 2.2.1. Cyclopentadepsipeptides

**Figure 3:**
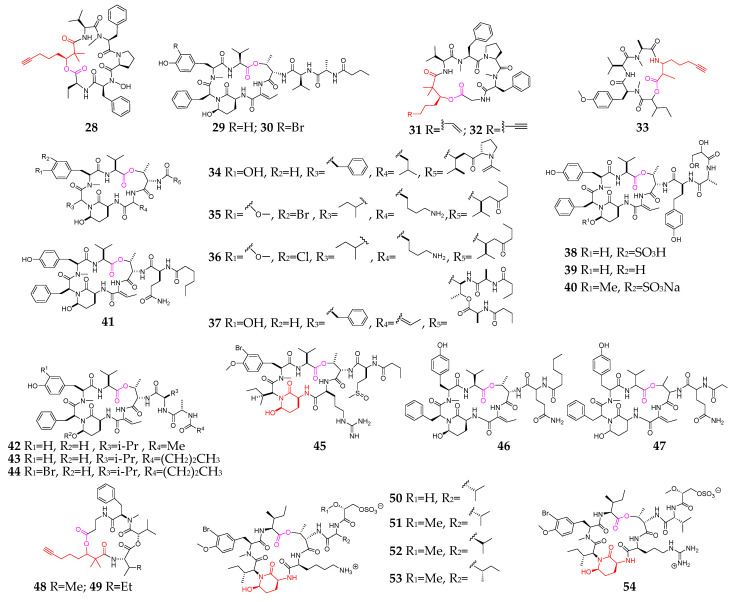

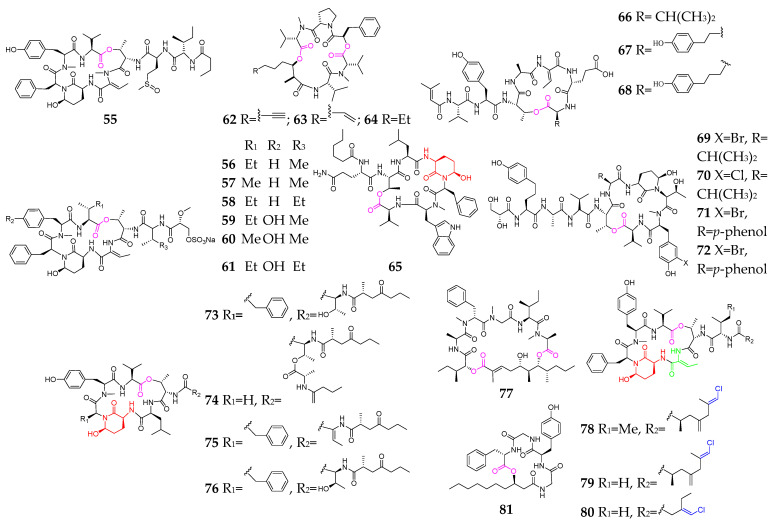
Marine-cyanobacterium-derived cyclopentadepsipeptides (**28**–**81**).

The marine cyanobacteria *Lyngbya*, *Symploca* and *Dichothrix* are the major sources of cyclopentadepsipeptides (**28**–**80**, Figure 3), of which compounds **28**, **32**, **33**, **48** and **49** contain a unique 2,2-dimethyl-3-hydroxy-7-octynoic acid (Dhoya) residue. Bouillomides A (**29**) and B (**30**) were reported as two new analogs of dolastatin 13 and showed selective inhibitory effects on the serine proteases elastase and chymotrypsin [30]. Cocosamides A (**31**) and B (**32**) exhibited weak cytotoxicities toward MCF7 and HT-29 cancer cells [31]. Guineamide C (**33**) possessed moderate cytotoxicity against a mouse neuroblastoma cell line [32]. Floridian *Lyngbya* sp.-derived novel cyclodepsipeptides (**34**–**36**) exhibited potent inhibitory effects on elastase, chymotrypsin and trypsin [33,34]. Kurahamide (**37**) was presented as a new dolastatin 13 analog and strongly inhibited elastase and chymotrypsin as well as showed moderately cytotoxic activity against HeLa and HL60 cells [35]. Lyngbyastatins 4–6 (**38**–**40**) contain an unusual amino acid homotyrosine residue and selectively inhibited porcine pancreatic elastase and chymotrypsin [36,37]. Lyngbyastatins 8–10 (**42**–**44**) had strong inhibitory effects on porcine pancreatic elastase with IC_50_ values of 123 nM, 210 nM and 120 nM, respectively [38].

Peptolide (**45**) and kyanamide (**65**) possess a 3-amino-6-hydroxy-2-piperidone (Ahp) residue, and the former could selectively inhibit trypsin over elastase and chymotrypsin with an IC_50_ value of 2.4 mM [39,40]. Jizanpeptins A−E (**50**–**54**), possessing an Ahp residue in a typical micropeptin motif, showed the specific inhibition of the serine protease trypsin in vitro and were cytotoxic to HeLa cervical and NCI-H460 lung cancer cell lines [41]. Structure–activity relationship (SAR) studies and X-ray cocrystal structure analysis suggested that compound **55** has similar activity, comparable to the clinically approved elastase inhibitor sivelestat in short-term assays and superior sustained activity in longer-term assays [42,43]. Trikoveramides A–C (**62**–**64**) are members of the kulolide superfamily [44]. Largamides A–G (**66**–**72**) are characterized by the unusual occurrence of a senecioic acid unit, and compounds **69**-**72** exhibited a strong inhibitory effect on chymotrypsin [45]. Loggerpeptins A–C (**73**–**75**) were reported as new Ahp-containing cyclic depsipeptides and displayed an inhibitory effect on the cleavage of the elastase substrate CD40 [46]. Molassamide (**76**) was the first depsipeptide reported from the marine cyanobacterial genus *Dichothrix* and has protease inhibitory activity [47]. Odoamide (**77**) showed potent cytotoxicity against HeLa S3 human cervical cancer cells with an IC_50_ value of 26.3 nM [48]. Tutuilamides A–C (**78**–**80**) are characterized by the presence of several unusual residues, including Ahp, 2-amino-2-butenoic acid and vinyl chloride [49].

#### 2.2.2. Cyclohexadepsipeptide

**Figure 4:**
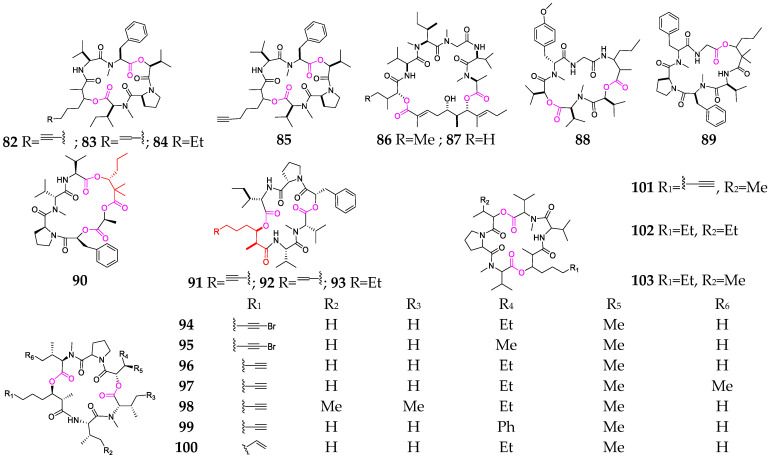
Marine-cyanobacterium-derived cyclohexadepsipeptides (**82**-**103**).

The marine cyanobacterium *Moorena producens* is the most important producer of cyclic hexadepsipeptides (**82**–**103**, Figure 4) [33,50,51,52,53,54,55]. The cytotoxicity-guided fractionation of a strain of *M. producens* collected from Papua New Guinea led to the isolation of aurilides B (**86**) and C (**87**), which showed excellent in vitro cytotoxicity toward NCI-H460 human lung tumor and the neuro-2a mouse neuroblastoma cell lines [51]. Guineamides D (**88**) and F (**89**) contain α-amino or α-hydroxy carboxylic acid residues and were isolated from a Papua New Guinea collection of *M. producens* [32]. Palmyramide A (**90**) features an unusual arrangement of three amino acids and three hydroxy acids; one of the hydroxy acids is the rare 2,2-dimethyl-3-hydroxyhexanoic acid (Dmhha) unit. This compound showed sodium-channel-blocking activity in neuro-2a cells and cytotoxic activity in H-460 human lung carcinoma cells [50]. Trungapeptins A–C (**91**–**93**) containing a 3-hydroxy-2-methyl-7-octynoic acid (Hmoya) residue were isolated and characterized from *M. producens* collected from Trung (Thailand) [52]. Veraguamides A-G (**94**–**100**) are characterized by the presence of an invariant proline residue, multiple *N*-methylated amino acids, an α-hydroxy acid and a C_8_-polyketide-derived β-hydroxy acid moiety with a characteristic terminus that is either an alkynyl bromide, alkyne or vinyl group. These metabolites showed moderate to weak cytotoxic activity against HT29 colorectal adenocarcinoma and HeLa cervical carcinoma cell lines [53].

#### 2.2.3. Cycloheptadepsipeptide

**Figure 5:**
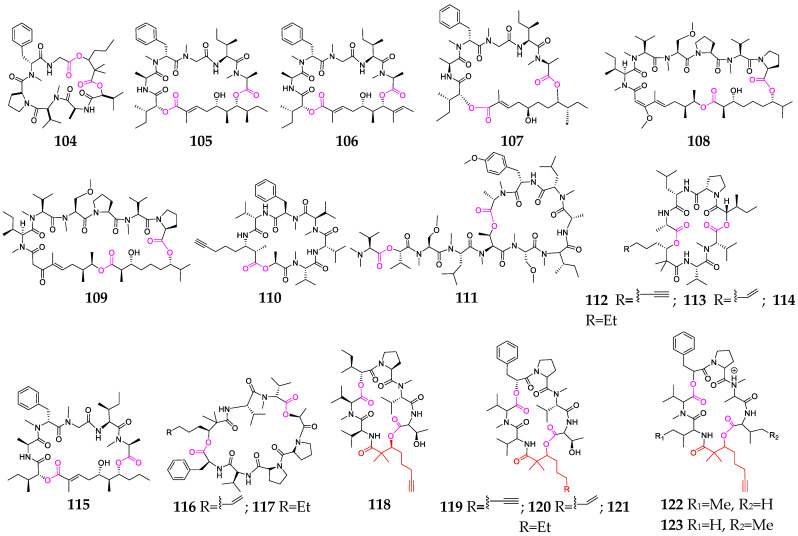
Marine-cyanobacterium-derived cycloheptadepsipeptides (**104**–**123**).

As of the end of 2022, as many as 20 cyclic heptadepsipeptides (**104**–**123**, Figure 5) had been isolated and characterized from marine cyanobacteria, including *Lyngbya*, *Leptolyngbya*, *Okeania*, *Dichothrix*, *Symploca* and *Rivularia* [32]. Compounds **104**–**110** were derived from several *Lyngbya* spp., and compounds **105**–**107** displayed significant antimalarial properties and potent cytotoxic activities against P388 murine leukemia cell lines. Kohamamides A–C (**112**–**114**), containing a Leu residue adjacent to a Pro residue, belong to the kulolide superfamily [54]. Lagunamide D (**115**) exhibited low-nanomolar antiproliferative activity against A549 human lung adenocarcinoma cells, while its structural transformation from a 26-membered macrocycle to a 24-membered ring structure led to a 9.6-fold decrease in activity [55]. Pemukainalides A (**116**) had cytotoxicity against the MOLT-4 leukemia cell line with an IC_50_ value of 5.6 μM [56]. Viequeamides A-F (**118**–**123**) are a family of 2,2-dimethyl-3-hydroxy-7-octanic acid-containing cyclic depsipeptides, and compound **118** was found to be highly toxic to H460 human lung cancer cells (IC_50_ = 60 ± 10 nM) [57].

#### 2.2.4. Thiazole-Containing Cyclodepsipeptides

**Figure 6:**
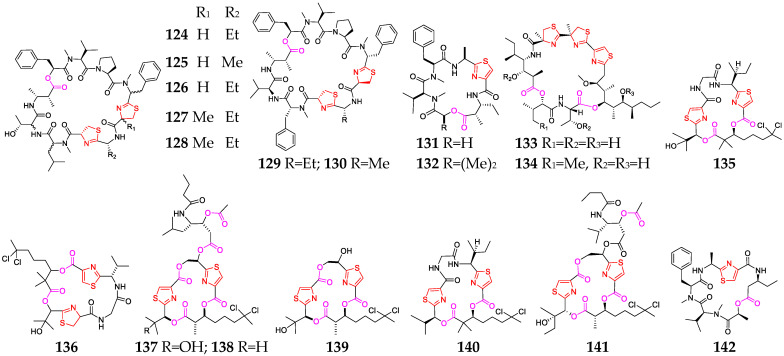
Marine-cyanobacterium-derived thiazole-containing cyclodepsipeptides (**124**–**142**).

Nineteen thiazole-containing cyclic depsipeptides (**124**–**142**, Figure 6) were discovered in three marine cyanobacteria: *Lyngbya*, *Leptolyngbya* and *Phormidium* [11,28,55,56,57,58,59,60]. Grassypeptolides A-G (**124**–**130**) are a group of closely related bis-thiazoline-containing cyclic depsipeptides. SAR analyses indicated that the ethyl substituent in **124** is changed to a methyl substituent in **125,** and its cytoactivity was only slightly reduced (3~4-fold), whereas the inversion of the Phe unit flanking the bis-thiazoline moiety resulted in 16~23-fold greater potency [58]. Compounds **127** and **128** showed significant cytotoxicity against HeLa and mouse neuro-2a blastoma cells [59], while **129** and **130** had moderate inhibitory activity against the transcription factor AP-1 (IC_50_ = 5.2 and 6.0 μM, respectively) [60]. Guineamides B (**132**) possessed moderate cytotoxicity against a mouse neuroblastoma cell line with an IC_50_ value of 15 µM [32]. Hoiamides A (**133**) and B (**134**) belong to the unique hoiamide structural class [15,61]. Compound **133** showed a potent inhibitory effect on [3H] batrachotoxin binding to voltage-gated sodium channels (IC_50_ = 92.8 nM) and activated sodium influx (EC_50_ = 2.31 μM) in mouse neocortical neurons, while **134** could stimulate sodium influx and suppressed spontaneous Ca^2+^ oscillations with EC_50_ values of 3.9 µM and 79.8 nM, respectively. Lyngbyabellin A (**135**) was shown to be a potent disrupter of the cellular microfilament network, and lyngbyabellin B (**136**) displayed potent toxicity toward brine shrimp and *Candida albicans* [62], while compounds **137** and **138** exhibited good cytotoxicity against NCI-H460 human lung tumor and neuro-2a mouse neuroblastoma cell lines, with LC_50_ values between 0.2 and 4.8 mM. Obyanamide (**142**), derived from a variety of *L. confervoides*, was cytotoxic against KB cells, with an IC_50_ value of 0.58 µg/mL [63].

#### 2.2.5. Other Cyclodepsipeptides

**Figure 7:**
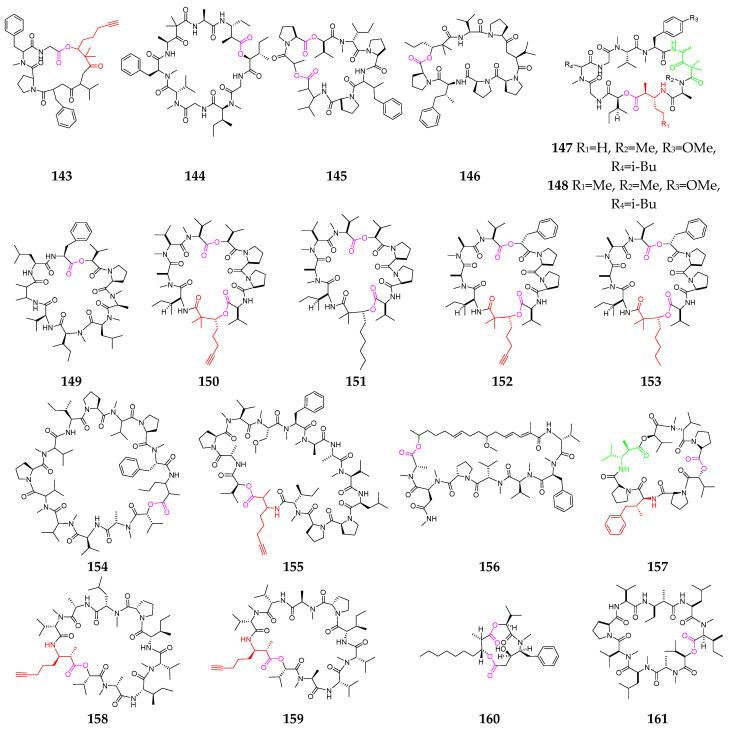
Marine-cyanobacterium-derived other cyclic depsipeptides (**143**–**161**).

Almost twenty other cyclodepsipeptides (**143**–**161**, Figure 7) have been obtained from *Lyngbya* [61,62,63,64,65,66], *Symploca* [67,68,69] and *Okeania* [70]. Guineamide G (**143**) showed potent brine shrimp toxicity and significant cytotoxicity against a mouse neuroblastoma cell line, with an LC_50_ value of 2.7 µM [64]. Desmethoxymajusculamide C (**145**) demonstrated potent and selective anti-solid-tumor activity against the HCT-116 human colon carcinoma cell line, with an IC_50_ value of 20 nM via the disruption of cellular microfilament networks [65]. Homodolastatin 16 (**146**), containing a 4-phenylvaline (dolaphenvaline, Dpv) moiety and a rare 2,2-dimethyl-3-hydroxyhexanoic acid (Dmhha) unit, shares higher homology with the potential anticancer agent dolastatin 16 [66]. Lyngbyastatin 3 (**148**) possesses two unusual amino acid residues, 3-amino-2-methylhexanoic acid (Amha) and 4-amino-2,2-dimethyl-3-oxopentanoic acid units (Ibu), and is a potent disrupter of cellular microfilament networks [67,68]. The four cytotoxic depsipeptides wewakpeptins A-D (**150**–**153**) represent an unusual arrangement of amino and hydroxy acid subunits and possess a bis-ester, a Dhoya or 2,2-dimethyl-3-hydroxyoctanoic acid (Dhoaa) residue, and a diprolyl group reminiscent of dolastatin 15 [69]. Malevamide E (**156**) had a potent inhibitory effect on Ca^2+^ release-activated Ca^2+^ (CRAC) channels [70]. Triproamide (**157**) contains the rare 4-phenylvaline (dolaphenvaline, Dpv) and a β-amino acid, dolamethylleucine (Dml), originally discovered in dolastatin 16 [56]. Companeramides A (**158**) and B (**159**) showed high nanomolar in vitro antiplasmodial activity [71], and hapalosin (**160**) displayed multidrug-resistance-reversing activity [72]. Urumamide (**161**) is a novel chymotrypsin inhibitor with a b-amino acid from a marine cyanobacterium *Okeania* sp. [73].

## 3. Marine Sponges

**Figure 8:**
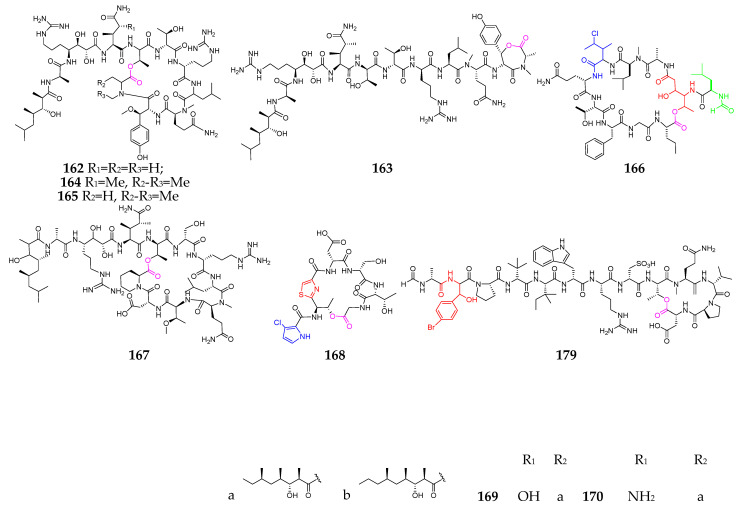

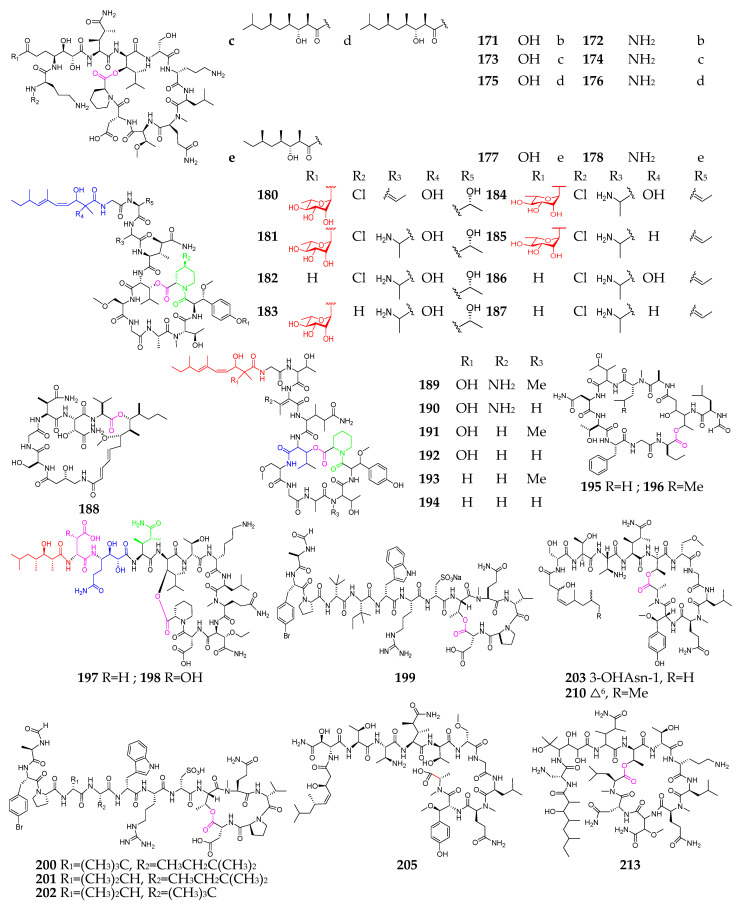

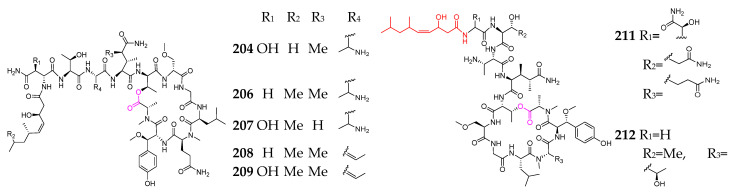
Marine-sponge-derived depsipeptides (**162**–**213**).

Marine sponges are well known as prolific sources of biologically natural products and are the second largest group of producers of marine-derived depsipeptides (**162**–**212**, Figure 8) [71,72,73,74,75,76,77,78,79,80,81,82,83,84]. With respect to the genus and species, however, there are no striking features about these sponges. Callipeltin A (**162**), obtained from a shallow water sponge of the genus *Callipelta*, exhibited a protective effect on cells infected with human immunodeficiency (HIV) virus [74]. Callipeltins N (**164**) and O (**165**) showed significant cytotoxicity against A2058, HT-29, MCF-7 and MRC-5 cell lines, with an IC_50_ value of 0.16 µM [75]. Cyclolithistide A (**166**) was discovered as a novel antifungal cyclodepsipeptide containing the unique amino acids 4-amino-3,5-dihydroxyhexanoic acid, formyl-leucine and chloroisoleucine [76]. Daedophamide (**167**) displayed strong cytotoxic activity against a panel of four human tumor cell lines with GI_50_ values in the submicromolar range [77]. Gunungamide A (**168**), produced by an Indonesian sponge *Discodermia* sp., possesses an unusual chloropyrrol ring [78]. Homophymine analogs (**169**–**178**) featuring new polyketide-derived end groups displayed potent antiproliferative activity (IC_50_ in the nM range) against a panel of human cancer cell lines [79,80]. Microspinosamide (**179**) was the first naturally occurring cyclodepsipeptide containing a ɑ-hydroxy-p-bromophenylalanine residue and inhibited the cytopathic effect of HIV-1 infection in an XTT-based in vitro assay, with an EC_50_ value of approximately 0.2 µg/mL [81]. Mirabamides A–H (**180**–**187**) have two new residues, 4-chlorohomoproline and β-methoxytyrosine 4′-O-α-*L*-rhamnopyranoside, along with a rare *N*-terminal aliphatic hydroxy acid, and were shown to potently inhibit HIV-1 fusion [82]. Papuamides A–F (**189**–**194**) were the first marine-derived depsipeptides reported to contain 3-hydroxyleucine and homoproline residues, as well as a previously undescribed 2,3-dihydroxy-2,6,8-trimethyldeca-(4*Z*,6*E*)-dienoic acid moiety. Both **189** and **190** inhibited the infection of human T-lymphoblastoid cells with HIV-1RF in vitro, with an EC_50_ of 4 ng/mL, and **189** was also cytotoxic against a panel of human cancer cell lines, with a mean IC_50_ value of 75 ng/mL [83]. Pipecolidepsins A and B (**197** and **198**) contain unusual residues, including 2-amino-3-hydroxy-4,5-dimethylhexanoic acid, 3-ethoxyasparagine,3,4-dimethylglutamine,4,7-diamino-2,3-dihydroxy-7-oxoheptanoic acid and 3-hydroxyaspartic acid, as well as a terminal 3-hydroxy-2,4,6-trimethylheptanoic acid residue [84]. Polydiscamides B–D (**200**–**202**) were the first examples of nonendogenous human SNSR agonists [85]. Two new HIV inhibitory depsipeptides, stellettapeptins A (**211**) and B (**212**), were the first peptides reported to contain a 3-hydroxy-6,8-dimethylnon-4-(*Z*)-enoic acid moiety [86]. Theopapuamide (**213**) was strongly cytotoxic against CEM-TART and HCT-116 cell lines, with EC_50_ values of 0.5 and 0.9 µM, respectively [87].

## 4. Marine Mollusks

**Figure 9:**
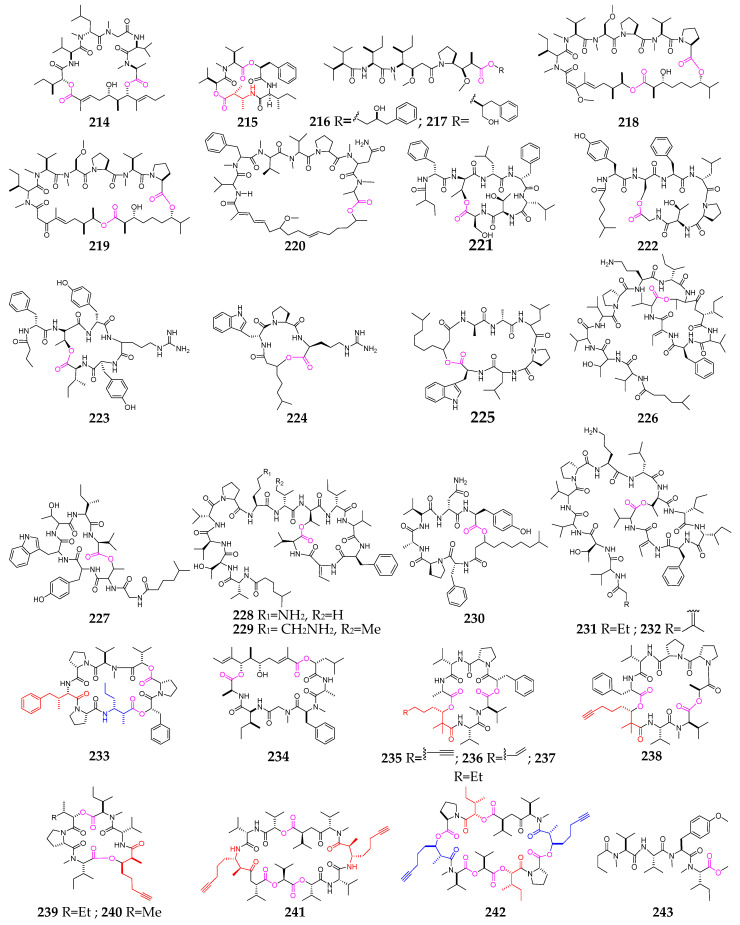
Marine-mollusk-derived depsipeptides (**214**–**243**).

At this point in time, a total of thirty depsipeptides (**214**–**243**, Figure 9) have been discovered in marine mollusks, including *Dolabella*, *Elysia*, *Philinopsis* and *Onchidium* [85,86,87,88,89,90,91,92,93,94]. Aurilide (**214**) was reported as a new 26-membered cyclodepsipeptide and displayed potent cytotoxicity against HeLa S3 cells, with an IC_50_ of 0.011 μg/mL [88]. Dolastatin D (**215**) is a cytotoxic cyclodepsipeptide possessing a novel β-amino acid (2*R*,3*R*)-3-amino-2-methylbutanoic acid residue [89]. Two 35-membered depsipeptides, dolastatin G (**218**) and nordolastatin G (**219**), exhibited cytotoxicity against HeLa S3 cells, with IC_50_ values of 1.0 and 5.3 µg/mL, respectively [90]. Dolastatin 14 (**220**), derived from the Indian Ocean sea hare *Dolabella auricularia*, was shown to be a novel cytostatic (PS EDm 0.022 μg/mL) agent [91]. Kahalalides Z_1_ (**231**) and Z_2_ (**232**) displayed potent antifungal properties and strong anticancer activities [92].

Two cytotoxic cyclodepsipeptides (**233** and **234**) containing two unusual amino acids, 4-phenylvaline and 3-amino-2-methylhexanoic acid, were purified from the cephalaspidean mollusk *Philinopsis speciosa* [93,94]. Kulolide, a cyclic depsipeptide, was isolated from a cephalaspidean mollusk, *Philinopsis speciosa* Pease [95], and kulolide (**235**), possessing a rare Dhoya residue, displayed a strong cytotoxic effect on L-1210 leukemia cells and P388 murine leukemia cells, with IC_50_ values of 0.7 and 2.1 µg/mL, respectively [95]. Onchidin A (**241**) was structurally determined to have a new *β*-amino acid, 3-amino-2-methyloct-7-ynoic acid (Amo) [96], while its analog onchidin B (**242**) contains two 2-hydroxy-3-methylpentanoic acid (Hmp) moieties and two 3-hydroxy-2-methyloct-7-ynoic acid (Hymo) units [97].

## 5. Marine Fungi

**Figure 10:**
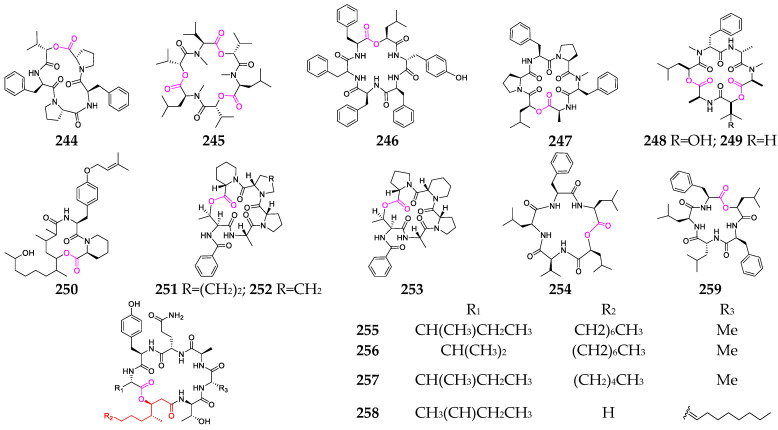
Marine-fungus-derived depsipeptides (**244**–**259**).

A growing body of evidence has indicated that marine fungi are important sources of depsipeptides, and the genus *Fusarium* is the most common producer. To date, all marine-fungus-derived depsipeptides (**244**–**259**, Figure 10) are cyclic and have a wide array of biological properties [95,96,97,98,99,100]. Enniatin G (**245**) has inhibitory activity against Heps 7402, with an ED_50_ value of 12 μg/mL [98]. Two cyclohexadepsipeptides, fusarihexin A (**246**) and fusarihexin B (**247**), are manufactured by the marine mangrove endophytic fungus *Fusarium* sp. R5 and exhibited stronger inhibitory activity against the plant pathogenic fungi C. *gloeosporioides*, C. *musae* and *F. oxysporum* than carbendazim, which is widely used as an agricultural and horticultural fungicide worldwide [99]. HA 23 (**250**) was reported as a novel cyclodepsipeptide containing a 14-carbon polyketide unit, a substituted tyrosine and pipecolinic acid [100]. Sansalvamide (**254**) exhibited selective in vitro cytotoxicity toward COLO 205 colon and SK-MEL-2 melanoma cancer cell lines [101]. W493 A-D (**255**-**258**) possess the unique residue 3-hydroxy-4-methyl-tetradecanoic acid (Hmta) and exhibited moderate activity against *Cladosporium cladosporiodes* and weak antitumor activity against the human ovarian cancer cell line A2780 [102,103]. Compound **261** had significant cytotoxicity in the NCI-60 cell line panel (median GI_50_ = 9.1μM), with highly enhanced selectivity against the CNS cancer cell line SF-268 (GI_50_ = 6.5 nM) and the renal cancer cell line RXF 393 (GI_50_ ≤ 5.0 nM) [75].

## 6. Marine Bacteria

**Figure 11:**
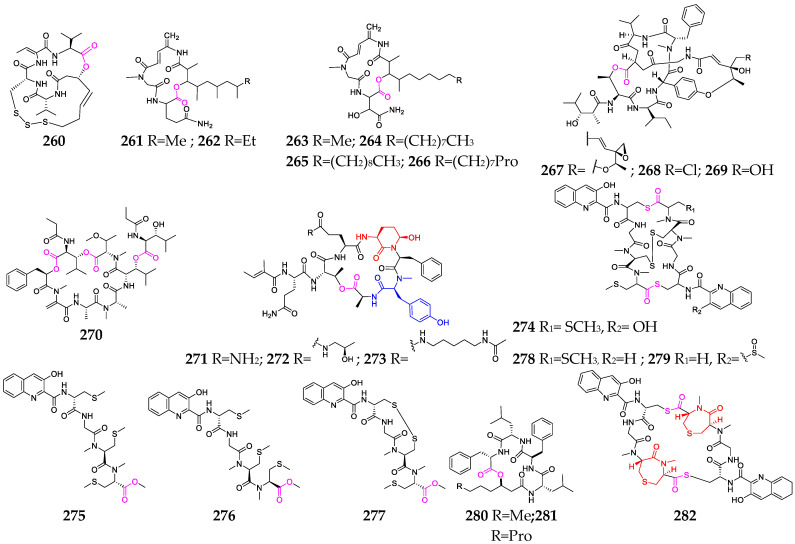
Marine-bacterium-derived depsipeptides (**260**–**282**).

Over twenty depsipeptides (**260**–**282**, Figure 11) have been identified in marine bacterial genera, including *Mciromonospora*, *Streptomyces*, *Chromobacterium*, *Verrucosispora* and *Photobacterium* [104]. Chromopeptide A (**260**) was investigated as a novel bicyclic depsipeptide and was found to suppress the proliferation of HL-60, K-562 and Ramos cells, with average IC_50_ values of 7.7, 7.0 and 16.5 nmol/L, respectively [105]. Rakicidins G–I (**264**–**266**), containing a long aliphatic chain without terminal methyl branching, were found to be 18.2~20.3-fold and 7.4~8.7-fold more cytotoxic under hypoxic than under normoxic conditions toward PANC-1 and HCT-8, respectively, and exhibited potent antibacterial effects against Gram-positive anaerobic bacteria [80]. Salinamides A (**267**) and B (**268**), sharing a rigid bicyclic hexadepsipeptide core with two esters and an aromatic ether link, showed significant topical anti-inflammatory activity [81]. Streptopeptolins A–C (**271**–**273**), containing the unusual amino acids Ahp and *N*-methyl tyrosine, were the first cyanopeptolin-type peptides isolated from *S. olivochromogenes* strain NBRC 3561 and demonstrated potent inhibitory activities against chymotrypsin [106]. Thiocoraline (**274**), produced by *Micromonospora* sp. strain L-13-ACM2-092, showed potent cytotoxic activity against P-388, A-549 and MEL-28 cell lines, as well as strong antimicrobial activity against Gram-positive bacteria by binding to supercoiling DNA and inhibiting RNA synthesis. Unnarmicins A (**280**) and C (**281**) selectively inhibited the growth of two strains belonging to the genus *Pseudovibrio*, one of the most prevalent genera in the marine environment [107]. Verrucosamide (**282**), composed of two rare seven-membered 1,4-thiazepane rings, was shown to have moderate cytotoxicity against MDA-MB-468 and COLO 205, with LD_50_ values of 1.26 µM and 1.4 µM, respectively [108].

## 7. Marine Algae

**Figure 12:**
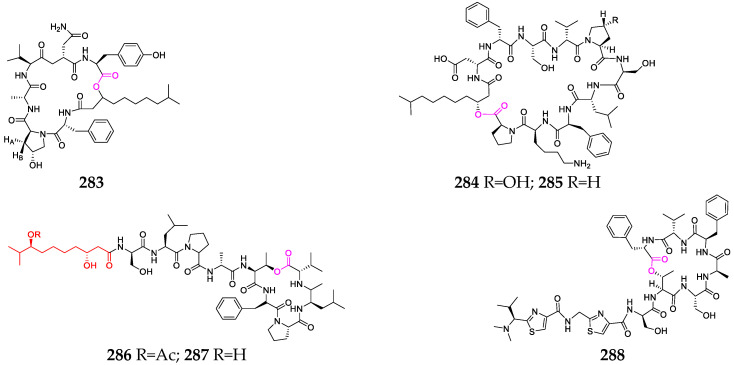
Marine-alga-derived depsipeptides (**283**–**288**).

To date, a half dozen depsipeptides (**283**–**288**, Figure 12) have been obtained from two marine macroalgae, *Bryopsis* and *Derbesia* [108]. Mebamamides A (**286**) and B (**287**) were reported as new lipopeptides with four D-amino acid residues and a 3,8-dihydroxy-9-methyldecanoic acid residue, but exhibited no growth inhibitory activity against HeLa and HL60 cells at 10 μM [109].

## 8. Conclusions and Perspectives

In summary, as many as 288 depsipeptides have been discovered in marine organisms, including cyanobacteria, sponges, mollusks, bacteria, fungi and algae, among which marine cyanobacteria are the largest group of producers. Most of these substances are formed by closing the loops of their terminal amino acids. It is very exciting that a large number of marine-derived cyclodepsipeptides display potent cytotoxic effects since they have absolute advantages in structural rigidity, biochemical stability, binding affinity and membrane permeability, which greatly improve their anticancer activity [110], such as the hormones or hormone analogs oxytocin [111], octreotide [112] and vasopressin [113], the antibiotics vancomycin [114], daptomycin [115] and polymyxin B [116] and the immunosuppressant cyclosporine [117]. Therefore, the discovery of novel marine cyclodepsipeptides for new drug development has been attractive to academic researchers and pharmaceutical companies. In the past decade, however, the number of new marine depsipeptides has been greatly reduced, as almost all accessible marine organisms have been collected and chemically studied. Fortunately, marine microorganisms (such as *Fusarium*, *Mciromonospora*, *Streptomyces*) have been shown to be a rich and unexploited source of bioactive natural products due to vast species richness and the biosynthetic potential of secondary metabolites, especially those of symbiotic microbes in marine sponges, mollusks, tunicates, macroalgae and mangroves. Therefore, more efforts should be made toward strain separation and chemical research using classical methods (e.g., strain cultivation and fermentation, chromatographic and spectroscopic techniques) and advanced approaches (e.g., metabolomics, genome mining and engineering).

## Data Availability

Not applicable.

## Supplementary Materials

The following supporting information can be downloaded at: https://www.mdpi.com/article/10.3390/md21020120/s1, Table S1: Detail information for marine cyanobacterium-derived depsipeptides (**1**-**161**); Table S2. Detail information for marine sponge-derived depsipeptides (**162**–**213**); Table S3: Detail information for marine mollusk-derived depsipeptides (**214**–**243**); Table S4 Detail information for marine fungus-derived depsipeptides (**244**–**259**); Table S5: Detail information for marine bacterium-derived depsipeptides (**260**–**282**); Table S6. Detail information for marine algae-derived depsipeptides (**283**–**288**).
