# Supplementary material for: Marine Organisms as a Prolific Source of Bioactive Depsipeptides
Source: Mar Drugs. 2023 Feb 11;21(2):120. doi: 10.3390/md21020120 (PMC9966715; doi:10.3390/md21020120)
Supplement: Supplementary file 1 [file marinedrugs-21-00120-s001.zip › marinedrugs-2202801-supplementary.pdf]

Supplementary Materials for

Marine organisms as a prolific source of bioactive depsipeptides

Mingyuan Zeng [1], Jianyun Tao [1] , Shuang Xu [1], Xuelian Bai [2] and Huawei Zhang [1,*]

[1] School of Pharmaceutical Sciences, Zhejiang University of Technology, Hangzhou 310014, China
[2] College of Life and Environmental Sciences, Hangzhou Normal University, Hangzhou 311121, China
* Correspondence: hwzhang@zjut.edu.cn

Contents

Table S1 Detail information for marine cyanobacterium-derived depsipeptides (**1-161**) S2
Table S2 Detail information for marine sponge-derived depsipeptides (**162-213**) S8
Table S3 Detail information for marine mollusk-derived depsipeptides (**214-243**) S10
Table S4 Detail information for marine fungus-derived depsipeptides (**244-259**) S11
Table S5 Detail information for marine bacterium-derived depsipeptides (**260-282**) S12
Table S6. Detail information for marine algae-derived depsipeptides (**283-288**). S13
References S14

Table S1 Marine cyanobacterium-derived depsipeptides (**1**-**161**).

Compound No.	Name	Biological Source/ Strain No.	Country/Region	Bioactivity	Ref.
1. Linear lipopeptide					
**1**	grassystatins D	*Symploca* VPG 14-61	Cetti Bay, Guam	potential antimetastatic activity against invasive breast cancer	[1]
**2**	grassystatins E				
**3**	grassystatins F				
**4**	hoiamide C	*Symploca* sp.	Gallows Reef, Papua New Guinea	-	[2]
**5**	hoiamide D		near Kape Point,Papua New Guinea	inhibitor of p53/MDM2 interaction	[3]
**6**	malevamides A	*Symploca laeteviridis*	south shore of Oahu, Hawaii	-	[4]
**7**	malevamide D	*Symploca hydnoides*		cytotoxic activity	[5]
**8**	symplostatin 3	*Symploca* sp. VP452	Kaneohe Bay, Oahu,Hawaii	vitro cytotoxicity activity	[6]
**9**	symplostatin 4	*Symploca* sp.	Key Largo (Florida Keys)	antimalarial activity	[7]
**10**	tasiamide A		Short Drop-off in Palau	cytotoxic activity	[8]
**11**	tasiamide B				[9]
**12**	tasiamide C		Kimbe Bay off the north coast of New Britain,Papua New Guinea	-	[10]
**13**	tasiamide D			-	
**14**	tasiamide E			-	
**15**	tasiamide F	*Lyngbya* sp. NIH code 399	Cocos Lagoon, Guam	vivo inhibitor of cathepsins D and E	[11]
**16**	izenamide A	*Lyngbya* 1605-5	Izena Island, Okinawa Prefecture, Japan	-	[12]
**17**	izenamide B			-	
**18**	izenamide C			-	
**19**	grassystatin A	*Lyngbya confervoides*	Key Largo of Florida	protease inhibitors activity	[13]
**20**	grassystatin B				
**21**	grassystatin C				
**22**	maedamide	*Lyngbya* sp.	Okinawa Prefecture	chymotrypsin inhibitor	[14]
**23**	lyngbyabellin D	*Lyngbya* sp VP417	Finger's Reef, Apra Harbor, Guam	against the KB cell line.	[15]
**24**	lyngbyabellin P	*Okeania* sp.	Red Sea	antifouling activity,	[16]
**25**	gallinamide A	*Schizothrix* sp	North coast of Panama	antimalarial and cytotoxic activity	[17]
**26**	veraguamide K	*Oscillatoria margaritifera* .	shallow water off Isla Canales de Afuera on the Pacific coast of Panama	cytotoxic activity	[18]
**27**	veraguamide L				
2 Cyclodepsipeptides					
2.1 Cyclopentadepsipeptides					

Compound No.	Name	Biological Source/ Strain No.	Country/Region	Bioactivity	Ref.
**28**	benderamide A	*Lyngbya* sp.	shores of St. John's Island, Singapore	-	[19]
**29**	bouillomide A	*Lyngbya bouillonii*	Guamanian	inhibition of serine proteases activity	[20]
**30**	bouillomide B	*Lyngbya bouillonii*	Guamanian	inhibition of serine proteases activity	[20]
**31**	cocosamide A	*Lyngbya majuscula*	Cocos Lagoon, Guam	weak cytotoxicity activity	[21]
**32**	cocosamide B	*Lyngbya majuscula*	Cocos Lagoon, Guam	weak cytotoxicity activity	[21]
**33**	guineamide C	*Lyngbya majuscula*	Papua New Guinea	cytotoxicity activity	[22]
**34**	kempopeptin A	*Lyngbya* sp	Kemp Channel in the Florida Keys	serine Protease Inhibitors activity	[23]
**35**	kempopeptin B	*Lyngbya* sp	Kemp Channel in the Florida Keys	serine Protease Inhibitors activity	[23]
**36**	kempopeptin C	*Lyngbya* sp	Kemp Channel in the Florida Keys	serine Protease Inhibitors activity	[24]
**37**	kurahamide	*Lyngbya* sp	Kuraha, Okinawa Prefecture, Japan	inhibited elastase and chymotrypsin activity	[25]
**38**	lyngbyastatin 4	*Lyngbya conferWoides*	Florida Atlantic coast	chymotrypsin inhibitor	[26]
**39**	lyngbyastatin 5	*Lyngbya* sp.	coast of Fort Lauderdale, Florida	selectively inhibit elastase	[27]
**40**	lyngbyastatin 6	*Lyngbya* sp.	coast of Fort Lauderdale, Florida	selectively inhibit elastase	[27]
**41**	lyngbyastatin 7	*Lyngbya* sp.	coast of Fort Lauderdale, Florida	selectively inhibit elastase	[27]
**42**	lyngbyastatin 8	*Lyngbya semiplena*	Tumon Bay, Guam	inhibit porcine pancreatic elastase	[28]
**43**	lyngbyastatin 9	*Lyngbya semiplena*	Tumon Bay, Guam	inhibit porcine pancreatic elastase	[28]
**44**	lyngbyastatin 10	*Lyngbya semiplena*	Tumon Bay, Guam	inhibit porcine pancreatic elastase	[28]
**45**	pompanopeptin A	*Lyngbya confervoides*	Near Fort Lauderdale, Florida, USA	selectively inhibited trypsin	[29]
**46**	somamide A	*Lyngbya majuscula* and *Schizothrix* sp.	near Somo , Fiji,and Taveuni Island, Fiji	-	[30]
**47**	somamide B	*Lyngbya majuscula* and *Schizothrix* sp.	near Somo , Fiji,and Taveuni Island, Fiji	-	[30]
**48**	yanucamide A	*Lyngbya majuscula* and *Schizothrix* sp.	Yanuca Island, Fiji	Brine shrimp toxicity	[31]
**49**	yanucamide B	*Lyngbya majuscula* and *Schizothrix* sp.	Yanuca Island, Fiji	Brine shrimp toxicity	[31]
**50**	jizanpeptin A	*Symploca* sp	Red Sea	protease Inhibitors activity	[32]
**51**	jizanpeptin B	*Symploca* sp	Red Sea	protease Inhibitors activity	[32]
**52**	jizanpeptin C	*Symploca* sp	Red Sea	protease Inhibitors activity	[32]
**53**	jizanpeptin D	*Symploca* sp	Red Sea	protease Inhibitors activity	[32]
**54**	jizanpeptin E	*Symploca* sp	Red Sea	protease Inhibitors activity	[32]
**55**	symplostatin 2	*Symploca hydnoides*	Pago Bay, Guam	-	[33]
**56**	symplostatin 5	*Symploca* sp.	Cetti Bay, Guam	elastase inhibitor activity	[34]
**57**	symplostatin 6	*Symploca* sp.	Cetti Bay, Guam	-	[34]
**58**	symplostatin 7	*Symploca* sp.	Cetti Bay, Guam	-	[34]
**59**	symplostatin 8	*Symploca* sp.	Cetti Bay, Guam	-	[34]
**60**	symplostatin 9	*Symploca* sp.	Cetti Bay, Guam	-	[34]
**61**	symplostatin 10	*Symploca* sp.	Cetti Bay, Guam	-	[34]
**62**	trikoveramide A	*Symploca hydnoides*	Bintan Island, Indonesia	cytotoxic activity	[35]

**63**	trikoveramide B				
**64**	trikoveramide C				
**65**	kyanamide	*Caldora penicillata*	Kyan, Okinawa Prefecture, Japan	protease inhibitory activity	[36]
**66**	largamides A	*Oscillatoria* sp.	Keys off the coast of Key Largo (Florida, USA)	-	[37]
**67**	largamides B	*Oscillatoria* sp.	Keys off the coast of Key Largo (Florida, USA)	-	[37]
**68**	largamides C	*Oscillatoria* sp.	Keys off the coast of Key Largo (Florida, USA)	-	[37]
**69**	largamides D	*Oscillatoria* sp.	Keys off the coast of Key Largo (Florida, USA)	inhibited chymotrypsin activity	[37]
**70**	largamides E	*Oscillatoria* sp.	Keys off the coast of Key Largo (Florida, USA)	inhibited chymotrypsin activity	[37]
**71**	largamides F	*Oscillatoria* sp.	Keys off the coast of Key Largo (Florida, USA)	inhibited chymotrypsin activity	[37]
**72**	largamides G	*Oscillatoria* sp.	Keys off the coast of Key Largo (Florida, USA)	inhibited chymotrypsin activity	[37]
**73**	loggerpeptin A	*Leptolyngbya* sp.	Loggerhead Key, Florida	-	[38]
**74**	loggerpeptin B	*Leptolyngbya* sp.	Loggerhead Key, Florida	-	[38]
**75**	loggerpeptin C	*Leptolyngbya* sp.	Loggerhead Key, Florida	protease Inhibition activity	[38]
**76**	molassamide	*Dichothrix utahensis*	Molasses Reef, Key Largo, Florida, and from Brewer's Bay, St. Thomas, U.S. Virgin Islands	protease-inhibitory activity	[39]
**77**	odoamide	*Okeania* sp.	Odo, Okinawa Prefecture, Japan	cytotoxic activity	[40]
**78**	tutuilamide A	-	American Samoa and Palmyra Atoll	elastase inhibitory activity	[41]
**79**	tutuilamide B	-	American Samoa and Palmyra Atoll	elastase inhibitory activity	[41]
**80**	tutuilamide C	-	American Samoa and Palmyra Atoll	elastase inhibitory activity	[41]
**81**	unnarmicin D	*Trichodesmium thiebautii*	Padre Island,Corpus Christi	-	[42]

2.2 Cycohexadepsipeptides

**Compound No.**	**Name**	**Biological Source/ Strain No.**	**Country/Region**	**Bioactivity**	**Ref.**
**82**	antanapeptin A	*Lyngbya majuscula*	Antany Mora, Madagascar	-	[43]
**83**	antanapeptin B	*Lyngbya majuscula*	Antany Mora, Madagascar	-	[43]
**84**	antanapeptin C	*Lyngbya majuscula*	Antany Mora, Madagascar	-	[43]
**85**	antanapeptin D	*Lyngbya majuscula*	Antany Mora, Madagascar	-	[43]
**86**	aurilides B	*Lyngbya majuscula*	Papua New Guinea	Cancer Cell Toxins	[44]
**87**	aurilides C	*Lyngbya majuscula*	Papua New Guinea	Cancer Cell Toxins	[44]
**88**	guineamide D	*Lyngbya majuscula*	Papua New Guinea	-	[22]
**89**	guineamide F	*Lyngbya majuscula*	Papua New Guinea	-	[22]
**90**	palmyramide A	*Lyngbya majuscula*	south of Strawn Island, Palmyra Atoll, USA	cytotoxic activity	[45]
**91**	trungapeptin A	*Lyngbya majuscula*	Thailand	-	[46]
**92**	trungapeptin B	*Lyngbya majuscula*	Trung Province, Thailand	-	[46]
**93**	trungapeptin C	*Lyngbya majuscula*	Trung Province, Thailand	-	[46]
**94**	veraguamide A	*Symploca* cf. *hydnoides*	Cetti Bay, Guam	cytotoxic activity	[47]

**95**	veraguamide B				
**96**	veraguamide C				
**97**	veraguamide D				
**98**	veraguamide E				
**99**	veraguamide F				
**100**	veraguamide G				
**101**	veraguamide H	*Oscillatoria margaritifera*	Coiba National Park, Panama	-	[18]
**102**	veraguamide I			-	
**103**	veraguamide J			-	

1.2.3 Cycloheptadepsipeptides

Compound No.	Name	Biological Source/ Strain No.	Country/Region	Bioactivity	Ref.
**104**	guineamide E	*Lyngbya majuscula*	Papua New Guinea	-	[22]
**105**	lagunamide A	*Lyngbya majuscula*	Pulau Hantu, Singapore	cytotoxic activity	[48]
**106**	lagunamide B				
**107**	lagunamide C		Pulau Hantu Besar Singapore		[49]
**108**	lyngbyastatin 2	*Lyngbya majuscula*	Finger's Reef, Apra Harbor,Guam	-	[50]
**109**	norlyngbyastatin 2			-	
**110**	ulongapeptin	*Lyngbya* sp	Palauan	cytotoxic activity	[51]
**111**	coibamide A	*Leptolyngbya* sp	Coiba National Park,Panama	Antiproliferative activity	[52]
**112**	kohamamide A	*Okeania* sp	Kohama Island, Okinawa Prefecture, Japan	-	[53]
**113**	kohamamide B			cytotoxicity activity	[53]
**114**	kohamamide C			-	
**115**	lagunamide D	*Dichothrix* sp	Loggerhead Key in the Dry Tortugas, Florida	cytotoxic activity	[54]
**116**	pemukainalide A	*Symploca hydnoides*	Trikora beach, Bintan Island	-	[55]
**117**	pemukainalide B			cytotoxic activity	
**118**	viequeamide A	*Rivularia* sp	Playa de la Chiva on Vieques Island in the Commonwealth of Puerto Rico, USA	cytotoxic activity	[56]
**119**	viequeamide B			-	
**120**	viequeamide C			-	
**121**	viequeamide D			-	
**122**	viequeamide E			-	
**123**	viequeamide F			-	

1.2.4 Thiazole-containing Cycodespipeptides

Compound No.	Name	Biological Source/ Strain No.	Country/Region	Bioactivity	Ref.
**124**	grassypeptolide A	*Lyngbya confervoides.*	Big Pine Shoals, Florida	Cytotoxicity activity	[57]
**125**	grassypeptolide B				
**126**	grassypeptolide C				

**127**	grassypeptolide D	*Leptolyngbya* sp	Red Sea		[58]
**128**	grassypeptolide E				
**129**	grassypeptolide F	*Lyngbya majuscula*	Ngerderrak Reef, Palau		[59]
**130**	grassypeptolide G				
**131**	guineamide A	*Lyngbya majuscula*	Papua New Guinea	-	[22]
**132**	guineamide B			Cytotoxicity activity	
**133**	hoiamide A	*Lyngbya majuscula*		Sodium Channel Activator	[60]
**134**	hoiamide B				
**135**	lyngbyabellin A	*Lyngbya majuscula*	Finger's Reef, Apra Harbor, Guam	cytotoxic activity	[61]
**136**	lyngbyabellin B		Bush Key, Dry Tortugas, FL	Antimicrobial activity	[62]
**137**	lyngbyabellin E		Alotau Bay, Papua New Guinea	cytotoxic activity	[63]
**138**	lyngbyabellin H				
**139**	lyngbyabellin G				
**140**	27-deoxylyngbyabellin A	*Lyngbya bouillonii*	Apra Harbor, Guam		[64]
**141**	lyngbyabellin J				
**142**	obyanamide	*Lyngbya confervoides*	Obyan Bay in Saipan	cytotoxic activity	[65]

1.2.5 Other Cyclodespipeptides

Compound No.	Name	Biological Source/ Strain No.	Country/Region	Bioactivity	Ref.
**143**	guineamide G	*Lyngbya majuscula*	Papua New Guinea	cytotoxicity activity	[66]
**144**	desmethoxymajusculamide C	*Lyngbya majuscula*	Yanuca Island, Fiji	cytotoxicity activity	[67]
**145**	homodolastatin 16		Wasini Island,Kenya	exhibited moderate activity	[68]
**146**	pitiprolamide	*Lyngbya majuscula*	Piti Bomb Holes, Guam	cytotoxic activity	[69]
**147**	lyngbyastatin 1	*Lyngbya majuscula/Schizothrix calcicola*	Guam Piti Bomb Hole	cytotoxic activity	[70]
**148**	lyngbyastatin 3	*Lyngbya majuscula*	Tokai Maru shipwreck in Apra Harbor, Guam	-	[71]
**149**	wewakamide A	*Lyngbya semiplena*	Papua New Guinea	brine shrimp toxicity	[66]
**150**	wewakpeptin A	*Lyngbya semiplena*	Papua New Guinea	cytotoxic activity	[72]
**151**	wewakpeptin B			cytotoxic activity	
**152**	wewakpeptin C			-	
**153**	wewakpeptin D			-	
**154**	malevamide B	*Symploca laete-viridis*	south shore of Oahu, Hawaii	-	[4]
**155**	malevamide C			-	
**156**	malevamide E		Oahu near Ala Moana Beach Park	inhibitory effect on Ca^{2+}	[73]
**157**	triproamide	*Symploca hydnoides*	Trikora beach, Bintan Island	-	[55]
**158**	companeramide A	*Symploca*	Coiba Island, Panama	vitro antiplasmodial activity	[74]
**159**	companeramide B				

**160**	hapalosin	*Hapalosiphon welwitschii*	-	multidrug-resistance reversing activity	[75]
**161**	urumamide	*Okeania* sp.	Ikei Island, Okinawa	inhibitory activity	[76]

Table S2 Detail information for marine sponge-derived depsipeptides (**162**-**213**).

Compound No.	Name	Biological Source/ Strain No.	Country/Region	Bioactivity	Ref.
**162**	callipeltin A	*Callipelta* sp.	New Caledonian Lithistida	Anti-HIV activity	[77]
**163**	callipeltin L	*Latrunculia* sp.	Vanuatu South Pacific	-	[78]
**164**	callipeltin N	*Asteropus* sp.	Mary Island, Russell group, Solomon Islands	cytotoxic activity	[79]
**165**	callipeltin O				
**166**	cyclolithistide A	*Theonella swinhoei.*	Jogashima, Sagami Bay,Japan	Antifungal activity	[80]
**167**	daedophamide	*Daedalopelta* sp.	Alor Island (Indonesia)	cytotoxic activity	[81]
**168**	gunungamide A	*Discodermia* sp.	Palau Gunung, Indonesia	-	[7]
**169**	homophymine A	*Homophymia* sp.	east coast of New Caledonia	Anti-HIV activity	[82]
**170**	homophymine A1	*Homophymia* sp.	New Caledonia	antiproliferative activity	[83]
**171**	homophymine B				
**172**	homophymine B2				
**173**	homophymine C				
**174**	homophymine C3				
**175**	homophymine D				
**176**	homophymine D4				
**177**	homophymine E				
**178**	homophymine E5				
**179**	microspinosamide	*Sidonops microspinosa*	near Sulawesi, Indonesia	Inhibited the cytopathic	[84]
**180**	mirabamide A	*Siliquariaspongia mirabilis*	Nama Island, southeast of Chuuk Lagoon, in the Federated States of Micronesia	Inhibit HIV-1 Fusion	[85]
**181**	mirabamide B				
**182**	mirabamide C				
**183**	mirabamide D				
**184**	mirabamide E	*Stelletta clavosa*	Torres Strait.		[86]
**185**	mirabamide F				
**186**	mirabamide G				
**187**	mirabamide H			Inhibit HIV-1 Fusion	[86]
**188**	nagahamide A	*Theonella swinhoei*	southern Japan	Antibacterial activity	[87]
**189**	papuamide A	*Theonella mirabilis* and *T. swinhoei*	near Madang Harbor on the north coast of Papua New Guinea	HIV-Inhibitory and Cytotoxic	[88]
**190**	papuamide B				
**191**	papuamide C			-	
**192**	papuamide D			-	
**193**	papuamide E	*Melophlus* sp.	Karumolum Russell Island in the Solomon Islands	cytotoxic activity	[89]
**194**	papuamide F				
**195**	phoriospongin A	*Phoriospongia* sp. and *Callyspongia bilamellata*	Indented Head	Nematocidal activity	[90]

**196**	phoriospongin B		Reef, Port Phillip Bay, Australia		
**197**	pipecolidepsin A	*Homophymia lamellosa*	near Saint Marie Island, Madagascar	cytotoxic activity	[91]
**198**	pipecolidepsin B				
**199**	polydiscamide A	*Discodermia* sp.	St. Lucia, Lesser Antilles	Inhibition of cell proliferation	[92]
**200**	polydiscamide B	*Ircinia* sp.	Porpoise Cay, the Great Barrier Reef, Australia	nonendogenous human SNSR agonists	[93]
**201**	polydiscamide C				
**202**	polydiscamide D				
**203**	stellatolide A	*Ecionemia acervus*	Madagascan	Cytotoxic Activity	[94]
**204**	stellatolide B			Cytotoxic Activity	
**205**	stellatolide C			Cytotoxic Activity	
**206**	stellatolide D				
**207**	stellatolide E				
**208**	stellatolide F				
**209**	stellatolide G				
**210**	stellatolide H	*Discodermia* sp.	East China Sea	cytotoxic activity	[95]
**211**	stellettapeptins A	*Stelletta* sp.	northwestern Australia	HIV-inhibitory	[96]
**212**	stellettapeptins B				
**213**	theopapuamide	*Theonella swinhoei*	Papua New Guinea	cytotoxic activity	[97]

Table S3 Detail information for marine mollusk-derived depsipeptides (**214**-**243**).

Compound No.	Name	Biological Source/ Strain No.	Country/Region	Bioactivity	Ref.
**214**	aurilide A	*Dolabella auricularia*	Shima Peninsula, Mie Prefecture, Japan	cytotoxic activity	[98]
**215**	dolastatin D			cytotoxic activity	[99]
**216**	dolastatin H				[100]
**217**	isodolastatin H				[100]
**218**	dolastatin G				[101]
**219**	nordolastatin G				
**220**	dolastatin 14		Indian Ocean	cytotoxic activity	[102]
**221**	kahalalide A	*Elysia rufescens*	Black Point, O’ahu	-	[103]
**222**	kahalalide B			-	
**223**	kahalalide C			-	
**224**	kahalalide D			-	
**225**	kahalalide E			-	
**226**	kahalalide F			Against AIDS OI Pathogens	[104]
**227**	kahalalide O	*Elysia ornata*		-	[105]
**228**	kahalalide R	*Elysia grandifolia*	Malvan in the west coast of India	-	[106]
**229**	kahalalide S			-	
**230**	kahalalide Y	*Elysia ornata*	Kahala Bay near Black Point, Oahu	-	[107]
**231**	kahalalide Z_1		coasts of Okha (India)	antfungal activity	[108]
**232**	kahalalide Z_2				
**233**	kulokekahilide-1	*Philinopsis speciosa*	Shark's Cove, Pupukea, O'ahu	cytotoxic activity	[109]
**234**	kulokekahilide-2			-	[110]
**235**	kulolide-1				[111]
**236**	kulolide-2			-	
**237**	kulolide-3			-	
**238**	kulokainallde-1			-	
**239**	kulomo’opunalide- 1			-	
**240**	kulomo’opunalide- 2			-	
**241**	onchidin A	*Onchidium* sp.	New Caledonia	-	[112]
**242**	onchidin B			-	[113]
**243**	pupukeamide	*Philinopsis* sp.	Shark's Cove, Pupukea, O'ahu	-	[114]

Table S4. Detail information for marine fungus-derived depsipeptides (**244**-**259**).

Compound No.	Name	Biological Source/ Strain No.	Country/Region	Bioactivity	Ref.
**244**	alternaramide	*Alternaria* sp. SF-5016	Masan Bay area, Korea	antibiotic activity	[115]
**245**	enniatin G	*Halosarpheia* sp. 732	South China Sea	inhibitory activity	[116]
**246**	fusarihexin A	*Fusarium* sp. R5	Leizhou Peninsula,China	antifungal activity	[117]
**247**	fusarihexin B				
**248**	guangomide A	Sponge-derived fungus strain no. 001314c	Guango, Papua New Guinea	cytotoxic activity	[118]
**249**	guangomide B				
**250**	HA23	*Fusarium* sp. (CANU-HA 23)	-	-	[119]
**251**	JBIR-113	*Penicillium* sp. fS36	Takarajima Island, Kagoshima Prefecture, Japan	-	[120]
**252**	JBIR-114			-	
**253**	JBIR-115			-	
**254**	sansalvamide	*Fusarium.* sp.	Little San Salvador Island, Bahamas	cytotoxic activity	[121]
**255**	W493 A	*Fusarium* sp	Shizuoka city in Jpan	antibacterial activity	[122]
**256**	W493 B				
**257**	W493 C		Chinese Mangrove plant Ceriops tagal	-	[123]
**258**	W493 D			-	
**259**	zygosporamide	*Zygosporium masonii*	Maui, Hawaii	cytotoxic activity	[124]

Table S5. Detail information for marine bacterium-derived depsipeptides (**260**-**282**).

Compound No.	Name	Biological Source/ Strain No.	Country/Region	Bioactivity	Ref.
**260**	chromopeptide A	*Chromobacterium* sp. HS-13-94	East China Sea	cytotoxic activity	[125]
**261**	rakicidin C	*Streptomyces* sp (strain GT 61042)	-	-	[126]
**262**	rakicidin D	*Streptomyces* sp. MWW064	Samut Sakhon province,Thailand	inhibitor of tumor cell invasion	[127]
**263**	rakicidin F	*Streptomyces* sp. GKU 220	Andaman sea, Ranong, Thailand	Antibacterial activity	[128]
**264**	rakicidin G	*Micromonospora chalcea* FIM 02-523	-	cytotoxic activity	[129]
**265**	rakicidin H		-		
**266**	rakicidin I		-		
**267**	salinamide A	*Streptomyces* sp. CNB-091	Florida Keys	antibiotic activity	[130]
**268**	salinamide B				
**269**	salinamide F			Antibiotic and inhibitor of bacterial RNA polymerase activity	[131]
**270**	sameuramide A	*Chromobacterum* sp. QS3666.	Sameura bay, Miyagi prefecture, Japan	-	[132]
**271**	streptopeptolin A	*Streptomyces olivochromogenes* NBRC 3561	Japan	inhibitory activity against chymotrypsin	[133]
**272**	streptopeptolin B			chymotrypsin inhibitory	[134]
**273**	streptopeptolin C			-	[134]
**274**	thiocoraline	*Micromonospora* sp L-13-ACM2-092	Indian Ocean near the coast of Mozambique.	Antitumor activity	[135]
**275**	thiochondrilline A	*Verrucosispora* sp.	Florida Keys	-	[136]
**276**	thiochondrilline B			-	
**277**	thiochondrilline C			cytotoxic activity	
**278**	22'-deoxythiocoraline				
**279**	12'-sulfoxythiocoraline				
**280**	unnarmicin A	*Photobacterium* sp. MBIC06485	Onna Beach, Okinawa, Japan	Antibacterial activity	[137]
**281**	unnarmicin C				
**282**	verrucosamide	*Verrucosispora* sp. CNX-026	Central West Coast of Florida	cytotoxic activity	[138]

Table S6. Detail information for marine algae-derived depsipeptides (**283**-**288**).

Compound No.	Name	Biological Source/ Strain No.	Country/Region	Bioactivity	Ref.
**283**	kahalalide K	*Bryopsis* sp.	Wai'anae Boat Harbor, Hawaii	-	[139]
**284**	kahalalide P			-	[140]
**285**	kahalalide Q			-	
**286**	mebamamide A	*Derbesia marina*	Mebama, Mie, Japan	induced the differentiation of HL60 cells into macrophage	[141]
**287**	mebamamide B				
**288**	pagoamide A	*Derbesia* sp.	Fagatele Bay, American Samoa	-	[142]

Reference

1. Al-Awadhi, F.H.; Law, B.K.; Paul, V.J.; Luesch, H. Grassystatins D-F, potent aspartic protease inhibitors from marine cyanobacteria as potential antimetastatic agents targeting invasive breast cancer. *J. Nat. Prod.* **2017**, *80*, 2969-2986, doi:10.1021/acs.jnatprod.7b00551.
2. Choi, H.; Pereira, A.R.; Cao, Z.; Shuman, C.F.; Engene, N.; Byrum, T.; Matainaho, T.; Murray, T.F.; Mangoni, A.; Gerwick, W.H. The hoiamides, structurally intriguing neurotoxic lipopeptides from Papua New Guinea marine cyanobacteria. *J. Nat. Prod.* **2010**, *73*, 1411-1421, doi:10.1021/np100468n.
3. Malloy, K.L.; Choi, H.; Fiorilla, C.; Valeriote, F.A.; Matainaho, T.; Gerwick, W.H. Hoiamide D, a marine cyanobacteria-derived inhibitor of p53/MDM2 interaction. *Bioorg. Med. Chem. Lett.* **2012**, *22*, 683-688, doi:10.1016/j.bmcl.2011.10.054.
4. Horgen, F.D.; Yoshida, W.Y.; Scheuer, P.J. Malevamides A-C, new depsipeptides from the marine cyanobacterium *Symploca* laete-viridis. *J. Nat. Prod.* **2000**, *63*, 461-467, doi:10.1021/np990449+.
5. Horgen, F.D.; Kazmierski, E.B.; Westenburg, H.E.; Yoshida, W.Y.; Scheuer, P.J. Malevamide D: isolation and structure determination of an isodolastatin H analogue from the marine cyanobacterium *Symploca hydnoides*. *J. Nat. Prod.* **2002**, *65*, 487-491, doi:10.1021/np010560r.
6. Luesch, H.; Yoshida, W.Y.; Moore, R.E.; Paul, V.J.; Mooberry, S.L.; Corbett, T.H. Symplostatin 3, a new dolastatin 10 analogue from the marine cyanobacterium *Symploca* sp. VP452. *J. Nat. Prod.* **2002**, *65*, 16-20, doi:10.1021/np010317s.
7. Tarazona, G.; Fernández, R.; Cruz, P.G.; Pérez, M.; Rodríguez, J.; Jiménez, C.; Cuevas, C. Combining JBCA and Marfey's methodology to determine the absolute configuration of threonines: the case of gunungamide A, a new cyclic depsipeptide containing chloropyrrole from the sponge *Discodermia* sp. *Org.Chem. Fron* **2019**, *6*, 15-21, doi:10.1039/c8qo00961a.
8. Williams, P.G.; Yoshida, W.Y.; Moore, R.E.; Paul, V.J. Tasiamide, a cytotoxic peptide from the marine cyanobacterium *Symploca* sp. *J. Nat. Prod.* **2002**, *65*, 1336-1339, doi:10.1021/np020184q.
9. Williams, P.G.; Yoshida, W.Y.; Moore, R.E.; Paul, V.J. The isolation and structure elucidation of Tasiamide B, a 4-amino-3-hydroxy-5-phenylpentanoic acid containing peptide from the marine cyanobacterium *Symploca* sp. *J. Nat. Prod.* **2003**, *66*, 1006-1009, doi:10.1021/np030114z.
10. Mevers, E.; Haeckl, F.P.; Boudreau, P.D.; Byrum, T.; Dorrestein, P.C.; Valeriote, F.A.; Gerwick, W.H. Lipopeptides from the tropical marine cyanobacterium *Symploca* sp. *J. Nat. Prod.* **2014**, *77*, 969-975, doi:10.1021/np401051z.
11. Al-Awadhi, F.H.; Ratnayake, R.; Paul, V.J.; Luesch, H. Tasiamide F, a potent inhibitor of cathepsins D and E from a marine cyanobacterium. *Bioorgan. Med. Chem.* **2016**, *24*, 3276-3282, doi:10.1016/j.bmc.2016.04.062.
12. Kanamori, Y.; Iwasaki, A.; Sumimoto, S.; Matsubara, T.; Sato, T.; Suenaga, K. Izenamides A and B, statine-containing depsipeptides, and an analogue from a marine

cyanobacterium. *J. Nat. Prod.* **2018**, *81*, 1673-1681, doi:10.1021/acs.jnatprod.8b00417.

13. Kwan, J.C.; Eksioglu, E.A.; Liu, C.; Paul, V.J.; Luesch, H. Grassystatins A-C from marine cyanobacteria, potent cathepsin E inhibitors that reduce antigen presentation. *J. Med. Chem.* **2009**, *52*, 5732-5747, doi:10.1021/jm9009394.
14. Iwasaki, A.; Ohno, O.; Sumimoto, S.; Suda, S.; Suenaga, K. Maedamide, a novel chymotrypsin inhibitor from a marine cyanobacterial assemblage of *Lyngbya* sp. *Tetrahedron Lett.* **2014**, *55*, 4126-4128, doi:10.1016/j.tetlet.2014.05.099.
15. Williams, P.G.; Luesch, H.; Yoshida, W.Y.; Moore, R.E.; Paul, V.J. Continuing studies on the cyanobacterium *Lyngbya* sp.: isolation and structure determination of 15-norlyngbyapeptin A and lyngbyabellin D. *J. Nat. Prod.* **2003**, *66*, 595-598, doi:10.1021/np030011g.
16. Petitbois, J.G.; Casalme, L.O.; Lopez, J.A.V.; Alarif, W.M.; Abdel-Lateff, A.; Al-Lihaibi, S.S.; Yoshimura, E.; Nogata, Y.; Umezawa, T.; Matsuda, F.; et al. Serinolamides and Lyngbyabellins from an *Okeania* sp. Cyanobacterium Collected from the Red Sea. *J. Nat. Prod.* **2017**, *80*, 2708-2715, doi:10.1021/acs.jnatprod.7b00449.
17. Linington, R.G.; Clark, B.R.; Trimble, E.E.; Almanza, A.; Ureña, L.D.; Kyle, D.E.; Gerwick, W.H. Antimalarial peptides from marine cyanobacteria: isolation and structural elucidation of gallinamide A. *J. Nat. Prod.* **2009**, *72*, 14-17, doi:10.1021/np8003529.
18. Mevers, E.; Liu, W.-T.; Engene, N.; Mohimani, H.; Byrum, T.; Pevzner, P.A.; Dorrestein, P.C.; Spadafora, C.; Gerwick, W.H. Cytotoxic veraguamides, alkynyl bromide-containing cyclic depsipeptides from the marine cyanobacterium cf. *Oscillatoria margaritifera*. *J. Nat. Prod.* **2011**, *74*, 928-936, doi:10.1021/np200077f.
19. Ding, C.Y.G.; Ong, J.F.M.; Goh, H.C.; Coffill, C.R.; Tan, L.T. Benderamide A, a cyclic depsipeptide from a singapore collection of marine cyanobacterium cf. *Lyngbya* sp. *Mar. Drugs* **2018**, *16*, 409, doi:10.3390/md16110409.
20. Rubio, B.K.; Parrish, S.M.; Yoshida, W.; Schupp, P.J.; Schils, T.; Williams, P.G. Depsipeptides from a guamanian marine cyanobacterium, *Lyngbya bouillonii*, with selective inhibition of serine proteases. *Tetrahedron Lett.* **2010**, *51*, 6718-6721, doi:10.1016/j.tetlet.2010.10.062.
21. Gunasekera, S.P.; Owle, C.S.; Montaser, R.; Luesch, H.; Paul, V.J. Malyngamide 3 and cocosamides A and B from the marine cyanobacterium *Lyngbya majuscula* from cocos lagoon, guam. *J. Nat. Prod.* **2011**, *74*, 871-876, doi:10.1021/np1008015.
22. Tan, L.T.; Sitachitta, N.; Gerwick, W.H. The guineamides, novel cyclic depsipeptides from a papua new guinea collection of the marine cyanobacterium *Lyngbya majuscula*. *J. Nat. Prod.* **2003**, *66*, 764-771, doi:10.1021/np020492o.
23. Taori, K.; Paul, V.J.; Luesch, H. Kempopeptins A and B, serine protease inhibitors with different selectivity profiles from a marine cyanobacterium, *Lyngbya* sp. *J. Nat. Prod.* **2008**, *71*, 1625-1629, doi:10.1021/np8002172.
24. Al-Awadhi, F.H.; Salvador, L.A.; Law, B.K.; Paul, V.J.; Luesch, H. Kempopeptin C, a novel marine-derived serine protease inhibitor targeting invasive breast cancer. *Mar. Drugs* **2017**, *15*, 290, doi:10.3390/md15090290.
25. Iwasaki, A.; Sumimoto, S.; Ohno, O.; Suda, S.; Suenaga, K. Kurahamide, a cyclic depsipeptide analog of dolastatin 13 from a marine cyanobacterial assemblage of

Lyngbya sp. *Bull. Chem. Soc. Jpn.* **2014**, *87*, 609-613, doi:10.1246/bcsj.20140008.

26. Matthew, S.; Ross, C.; Rocca, J.R.; Paul, V.J.; Luesch, H. Lyngbyastatin 4, a dolastatin 13 analogue with elastase and chymotrypsin inhibitory activity from the marine cyanobacterium *Lyngbya confervoides*. *J. Nat. Prod.* **2007**, *70*, 124-127, doi:10.1021/np060471k.
27. Taori, K.; Matthew, S.; Rocca, J.R.; Paul, V.J.; Luesch, H. Lyngbyastatins 5-7, potent elastase inhibitors from floridian marine cyanobacteria, *Lyngbya* spp. *J. Nat. Prod.* **2007**, *70*, 1593-1600, doi:10.1021/np0702436.
28. Kwan, J.C.; Taori, K.; Paul, V.J.; Luesch, H. Lyngbyastatins 8-10, elastase inhibitors with cyclic depsipeptide scaffolds isolated from the marine cyanobacterium *Lyngbya semiplena*. *Mar. Drugs* **2009**, *7*, 528-538, doi:10.3390/md7040528.
29. Matthew, S.; Ross, C.; Paul, V.J.; Luesch, H. Pompanopeptins A and B, new cyclic peptides from the marine cyanobacterium *Lyngbya confervoides*. *Tetrahedron* **2008**, *64*, 4081-4089, doi:10.1016/j.tet.2008.02.035.
30. Nogle, L.M.; Williamson, R.T.; Gerwick, W.H. Somamides A and B, two new depsipeptide analogues of dolastatin 13 from a Fijian cyanobacterial assemblage of *Lyngbya majuscula* and *Schizothrix* species. *J. Nat. Prod.* **2001**, *64*, 716-719, doi:10.1021/np000634j.
31. Sitachitta, N.; Williamson, R.T.; Gerwick, W.H. Yanucamides A and B, two new depsipeptides from an assemblage of the marine cyanobacteria *Lyngbya majuscula* and *Schizothrix* species. *J. Nat. Prod.* **2000**, *63*, 197-200, doi:10.1021/np990466z.
32. Gallegos, D.A.; Sauri, J.; Cohen, R.D.; Wan, X.; Videau, P.; Vallota-Eastman, A.O.; Shaala, L.A.; Youssef, D.T.A.; Williamson, R.T.; Martin, G.E.; et al. Jizanpeptins, cyanobacterial protease inhibitors from a *Symploca* sp. cyanobacterium collected in the red sea. *J. Nat. Prod.* **2018**, *81*, 1417-1425, doi:10.1021/acs.jnatprod.8b00117.
33. Harrigan, G.G.; Luesch, H.; Yoshida, W.Y.; Moore, R.E.; Nagle, D.G.; Paul, V.J. Symplostatin 2: a dolastatin 13 analogue from the marine cyanobacterium *Symploca hydnoides*. *J. Nat. Prod.* **1999**, *62*, 655-658, doi:10.1021/np980553b.
34. Salvador, L.A.; Taori, K.; Biggs, J.S.; Jakoncic, J.; Ostrov, D.A.; Paul, V.J.; Luesch, H. Potent elastase inhibitors from cyanobacteria: structural basis and mechanisms mediating cytoprotective and anti-inflammatory effects in bronchial epithelial cells. *J. Med. Chem.* **2013**, *56*, 1276-1290, doi:10.1021/jm3017305.
35. Phyo, M.Y.; Katermeran, N.P.; Goh, J.X.; Tan, L.T. Trikoveramides A-C, cyclic depsipeptides from the marine cyanobacterium *Symploca hydnoides*. *Phytochemistry* **2021**, *190*, 112879, doi:10.1016/j.phytochem.2021.112879.
36. Ozaki, K.; Iwasaki, A.; Suenaga, K.; Teruya, T. Kyanamide, a new Ahp-containing depsipeptide from marine cyanobacterium *Caldora* penicillata. *Tetrahedron* **2019**, *75*, 3382-3386, doi:10.1016/j.tet.2019.04.046.
37. Plaza, A.; Bewley, C.A. Largamides A-H, unusual cyclic peptides from the marine cyanobacterium *Oscillatoria* sp. *J. Org. Chem.* **2006**, *71*, 6898-6907, doi:10.1021/jo061044e.

38. Al-Awadhi, F.H.; Paul, V.J.; Luesch, H. Structural diversity and anticancer activity of marine-derived elastase inhibitors: Key features and mechanisms mediating the antimetastatic effects in invasive breast cancer. *Chembiochem* **2018**, *19*, 815-825, doi:10.1002/cbic.201700627.
39. Gunasekera, S.P.; Miller, M.W.; Kwan, J.C.; Luesch, H.; Paul, V.J. Molassamide, a depsipeptide serine protease inhibitor from the marine cyanobacterium *Dichothrix utahensis*. *J. Nat. Prod.* **2010**, *73*, 459-462, doi:10.1021/np900603f.
40. Sueyoshi, K.; Kaneda, M.; Sumimoto, S.; Oishi, S.; Fujii, N.; Suenaga, K.; Teruya, T. Odoamide, a cytotoxic cyclodepsipeptide from the marine cyanobacterium *Okeania* sp. *Tetrahedron* **2016**, *72*, 5472-5478, doi:10.1016/j.tet.2016.07.031.
41. Keller, L.; Canuto, K.M.; Liu, C.; Suzuki, B.M.; Almaliti, J.; Sikandar, A.; Naman, C.B.; Glukhov, E.; Luo, D.; Duggan, B.M.; et al. Tutuilamides A-C: vinyl-chloride-containing cyclodepsipeptides from marine cyanobacteria with potent elastase inhibitory properties. *ACS Chem. Biol.* **2020**, *15*, 751-757, doi:10.1021/acschembio.9b00992.
42. Bertin, M.J.; Roduit, A.F.; Sun, J.; Alves, G.E.; Via, C.W.; Gonzalez, M.A.; Zimba, P.V.; Moeller, P.D.R. Tricholides A and B and Unnarmicin D: new hybrid PKS-NRPS macrocycles isolated from an environmental collection of *Trichodesmium thiebautii*. *Mar. Drugs* **2017**, *15*, 206, doi:10.3390/md15070206.
43. Nogle, L.M.; Gerwick, W.H. Isolation of four new cyclic depsipeptides, antanapeptins A-D, and dolastatin 16 from a Madagascan collection of *Lyngbya majuscula*. *J. Nat. Prod.* **2002**, *65*, 21-24, doi:10.1021/np010348n.
44. Han, B.; Gross, H.; Goeger, D.E.; Mooberry, S.L.; Gerwick, W.H. Aurilides B and C, cancer cell toxins from a papua new guinea collection of the marine cyanobacterium *Lyngbya majuscula*. *J. Nat. Prod.* **2006**, *69*, 572-575, doi:10.1021/np0503911.
45. Taniguchi, M.; Nunnery, J.K.; Engene, N.; Esquenazi, E.; Byrum, T.; Dorrestein, P.C.; Gerwick, W.H. Palmyramide A, a cyclic depsipeptide from a Palmyra Atoll collection of the marine cyanobacterium *Lyngbya majuscula*. *J. Nat. Prod.* **2010**, *73*, 393-398, doi:10.1021/np900428h.
46. Bunyajetpong, S.; Yoshida, W.Y.; Sitachitta, N.; Kaya, K. Trungapeptins A-C, cyclodepsipeptides from the marine cyanobacterium *Lyngbya majuscula*. *J. Nat. Prod.* **2006**, *69*, 1539-1542, doi:10.1021/np050485a.
47. Salvador, L.A.; Biggs, J.S.; Paul, V.J.; Luesch, H. Veraguamides A-G, cyclic hexadepsipeptides from a dolastatin 16-producing cyanobacterium *Symploca* cf. hydnoides from Guam. *J. Nat. Prod.* **2011**, *74*, 917-927, doi:10.1021/np200076t.
48. Tripathi, A.; Puddick, J.; Prinsep, M.R.; Rottmann, M.; Tan, L.T. Lagunamides A and B: cytotoxic and antimalarial cyclodepsipeptides from the marine cyanobacterium *Lyngbya majuscula*. *J. Nat. Prod.* **2010**, *73*, 1810-1814, doi:10.1021/np100442x.
49. Tripathi, A.; Puddick, J.; Prinsep, M.R.; Rottmann, M.; Chan, K.P.; Chen, D.Y.-K.; Tan, L.T. Lagunamide C, a cytotoxic cyclodepsipeptide from the marine cyanobacterium *Lyngbya majuscula*. *Phytochemistry* **2011**, *72*, 2369-2375, doi:10.1016/j.phytochem.2011.08.019.
50. Luesch, H.; Yoshida, W.Y.; Moore, R.E.; Paul, V.J. Lyngbyastatin 2 and norlyngbyastatin 2, analogues of dolastatin G and nordolastatin G from the marine

cyanobacterium *Lyngbya majuscula*. *J. Nat. Prod.* **1999**, *62*, 1702-1706, doi:10.1021/np990310z.

51. Williams, P.G.; Yoshida, W.Y.; Quon, M.K.; Moore, R.E.; Paul, V.J. Ulongapeptin, a cytotoxic cyclic depsipeptide from a Palauan marine cyanobacterium *Lyngbya* sp. *J. Nat. Prod.* **2003**, *66*, 651-654, doi:10.1021/np030050s.
52. Medina, R.A.; Goeger, D.E.; Hills, P.; Mooberry, S.L.; Huang, N.; Romero, L.I.; Ortega-Barria, E.; Gerwick, W.H.; McPhail, K.L. Coibamide A, a potent antiproliferative cyclic depsipeptide from the Panamanian marine cyanobacterium *Leptolyngbya* sp. *J. Am. Chem. Soc.* **2008**, *130*, 6324-6325, doi:10.1021/ja801383f.
53. Iwasaki, A.; Shiota, I.; Sumimoto, S.; Matsubara, T.; Sato, T.; Suenaga, K. Kohamamides A, B, and C, cyclic depsipeptides from an *Okeania* sp. marine cyanobacterium. *J. Nat. Prod.* **2017**, *80*, 1948-1952, doi:10.1021/acs.jnatprod.7b00256.
54. Luo, D.; Putra, M.Y.; Ye, T.; Paul, V.J.; Luesch, H. Isolation, structure elucidation and biological evaluation of Lagunamide D: a new cytotoxic macrocyclic depsipeptide from marine cyanobacteria. *Mar. Drugs* **2019**, *17*, 83, doi:10.3390/md17020083.
55. Phyo, M.Y.; Goh, J.X.; Tan, L.T. Triproamide and Pemukainalides, cyclic depsipeptides from the marine cyanobacterium *Symploca hydnoides*. *J. Nat. Prod.* **2022**, *85*, 485-492, doi:10.1021/acs.jnatprod.1c00996.
56. Boudreau, P.D.; Byrum, T.; Liu, W.T.; Dorrestein, P.C.; Gerwick, W.H. Viequeamide A, a cytotoxic member of the kulolide superfamily of cyclic depsipeptides from a marine button cyanobacterium. *J. Nat. Prod.* **2012**, *75*, 1560-1570, doi:10.1021/np300321b.
57. Kwan, J.C.; Ratnayake, R.; Abboud, K.A.; Paul, V.J.; Luesch, H. Grassypeptolides A-C, cytotoxic bis-thiazoline containing marine cyclodepsipeptides. *J. Org. Chem.* **2010**, *75*, 8012-8023, doi:10.1021/jo1013564.
58. Thornburg, C.C.; Thimmaiah, M.; Shaala, L.A.; Hau, A.M.; Malmo, J.M.; Ishmael, J.E.; Youssef, D.T.; McPhail, K.L. Cyclic depsipeptides, grassypeptolides D and E and Ibu-epidemethoxylyngbyastatin 3, from a Red Sea *Leptolyngbya* cyanobacterium. *J. Nat. Prod.* **2011**, *74*, 1677-1685, doi:10.1021/np200270d.
59. Popplewell, W.L.; Ratnayake, R.; Wilson, J.A.; Beutler, J.A.; Colburn, N.H.; Henrich, C.J.; McMahon, J.B.; McKee, T.C. Grassypeptolides F and G, cyanobacterial peptides from *Lyngbya majuscula*. *J. Nat. Prod.* **2011**, *74*, 1686-1691, doi:10.1021/np2005083.
60. Pereira, A.; Cao, Z.; Murray, T.F.; Gerwick, W.H. Hoiamide a, a sodium channel activator of unusual architecture from a consortium of two papua new Guinea cyanobacteria. *Chem. Biol.* **2009**, *16*, 893-906, doi:10.1016/j.chembiol.2009.06.012.
61. Luesch, H.; Yoshida, W.Y.; Moore, R.E.; Paul, V.J.; Mooberry, S.L. Isolation, structure determination, and biological activity of Lyngbyabellin A from the marine cyanobacterium *lyngbya majuscula*. *J. Nat. Prod.* **2000**, *63*, 611-615, doi:10.1021/np990543q.
62. Milligan, K.E.; Marquez, B.L.; Williamson, R.T.; Gerwick, W.H. Lyngbyabellin B, a toxic and antifungal secondary metabolite from the marine cyanobacterium *Lyngbya majuscula*. *J. Nat. Prod.* **2000**, *63*, 1440-1443, doi:10.1021/np000133y.
63. Han, B.; McPhail, K.L.; Gross, H.; Goeger, D.E.; Mooberry, S.L.; Gerwick, W.H. Isolation and structure of five lyngbyabellin derivatives from a papua new guinea

collection of the marine cyanobacterium *Lyngbya majuscula*. *Tetrahedron* **2005**, *61*, 11723-11729, doi:10.1016/j.tet.2005.09.036.

64. Matthew, S.; Salvador, L.A.; Schupp, P.J.; Paul, V.J.; Luesch, H. Cytotoxic halogenated macrolides and modified peptides from the apratoxin-producing marine cyanobacterium *Lyngbya bouillonii* from Guam. *J. Nat. Prod.* **2010**, *73*, 1544-1552, doi:10.1021/np1004032.
65. Williams, P.G.; Yoshida, W.Y.; Moore, R.E.; Paul, V.J. Isolation and structure determination of obyanamide, a novel cytotoxic cyclic depsipeptide from the marine cyanobacterium *Lyngbya* confervoides. *J. Nat. Prod.* **2002**, *65*, 29-31, doi:10.1021/np0102253.
66. Han, B.; Gross, H.; McPhail, K.L.; Goeger, D.; Maier, C.S.; Gerwick, W.H. Wewakamide A and guineamide G, cyclic depsipeptides from the marine cyanobacteria *Lyngbya semiplena* and *Lyngbya majuscula*. *J. Mic.Biote.* **2011**, *21*, 930-936, doi:10.4014/jmb.1105.05011.
67. Simmons, T.L.; Nogle, L.M.; Media, J.; Valeriote, F.A.; Mooberry, S.L.; Gerwick, W.H. Desmethoxymajusculamide C, a cyanobacterial depsipeptide with potent cytotoxicity in both cyclic and ring-opened forms. *J. Nat. Prod.* **2009**, *72*, 1011-1016, doi:10.1021/np9001674.
68. Davies-Coleman, M.T.; Dzeha, T.M.; Gray, C.A.; Hess, S.; Pannell, L.K.; Hendricks, D.T.; Arendse, C.E. Isolation of homodolastatin 16, a new cyclic depsipeptide from a kenyan collection of *Lyngbya majuscula*. *J. Nat. Prod.* **2003**, *66*, 712-715, doi:10.1021/np030014t.
69. Montaser, R.; Abboud, K.A.; Paul, V.J.; Luesch, H. Pitiprolamide, a proline-rich dolastatin 16 analogue from the marine cyanobacterium *Lyngbya majuscula* from Guam. *J. Nat. Prod.* **2011**, *74*, 109-112, doi:10.1021/np1006839.
70. Harrigan, G.G.; Yoshida, W.Y.; Moore, R.E.; Nagle, D.G.; Park, P.U.; Biggs, J.; Paul, V.J.; Mooberry, S.L.; Corbett, T.H.; Valeriote, F.A. Isolation, structure determination, and biological activity of dolastatin 12 and lyngbyastatin 1 from *Lyngbya majuscula*/*Schizothrix calcicola* cyanobacterial assemblages. *J. Nat. Prod.* **1998**, *61*, 1221-1225, doi:10.1021/np9801211.
71. Williams, P.G.; Moore, R.E.; Paul, V.J. Isolation and structure determination of lyngbyastatin 3, a lyngbyastatin 1 homologue from the marine cyanobacterium *Lyngbya majuscula*. Determination of the configuration of the 4-amino-2,2-dimethyl-3-oxopentanoic acid unit in majusculamide C, dolastatin 12, lyngbyastatin 1, and lyngbyastatin 3 from cyanobacteria. *J. Nat. Prod.* **2003**, *66*, 1356-1363, doi:10.1021/np0302145.
72. Han, B.; Goeger, D.; Maier, C.S.; Gerwick, W.H. The wewakpeptins, cyclic depsipeptides from a papua new guinea collection of the marine cyanobacterium *Lyngbya semiplena*. *J. Org. Chem.* **2005**, *70*, 3133-3139, doi:10.1021/jo0478858.
73. Adams, B.; Porzgen, P.; Pittman, E.; Yoshida, W.Y.; Westenburg, H.E.; Horgen, F.D. Isolation and structure determination of malevamide E, a dolastatin 14 analogue, from the marine cyanobacterium *symploca laete-viridis*. *J. Nat. Prod.* **2008**, *71*, 750-754, doi:10.1021/np070346o.
74. Vining, O.B.; Medina, R.A.; Mitchell, E.A.; Videau, P.; Li, D.; Serrill, J.D.; Kelly, J.X.; Gerwick, W.H.; Proteau, P.J.; Ishmael, J.E.; et al. Depsipeptide companeramides from a Panamanian marine cyanobacterium associated with the coibamide producer. *J. Nat. Prod.* **2015**, *78*, 413-420, doi:10.1021/np5007907.
75. Stratmann, K.; Burgoyne, D.L.; Moore, R.E.; Patterson, G.M.L.; Smith, C.D. Hapalosin, a cyanobacterial cyclic depsipeptide with multidrug-resistance reversing activity.

J. Org. Chem. **2002**, *59*, 7219-7226, doi:10.1021/jo00103a011.

76. Kanamori, Y.; Iwasaki, A.; Sumimoto, S.; Suenaga, K. Urumamide, a novel chymotrypsin inhibitor with a β-amino acid from a marine cyanobacterium *Okeania* sp. *Tetrahedron Lett.* **2016**, *57*, 4213-4216, doi:10.1016/j.tetlet.2016.08.012.
77. Zampella, A.; D'Auria, M.V.; Paloma, L.G.; Casapullo, A.; Minale, L.; Debitus, C.; Henin, Y. Callipeltin A, an anti-HIV cyclic depsipeptide from the new caledonian lithistida sponge *Callipelta* sp. *J. Am. Chem. Soc.* **1996**, *118*, 6202-6209, doi:10.1021/ja954287p.
78. D'Auria, M.V.; Sepe, V.; D'Orsi, R.; Bellotta, F.; Debitus, C.; Zampella, A. Isolation and structural elucidation of callipeltins J–M: antifungal peptides from the marine sponge *Latrunculia* sp. *Tetrahedron* **2007**, *63*, 131-140, doi:10.1016/j.tet.2006.10.032.
79. Stierhof, M.; Hansen, K.Ø.; Sharma, M.; Feussner, K.; Subko, K.; Díaz-Rullo, F.F.; Isaksson, J.; Pérez-Victoria, I.; Clarke, D.; Hansen, E.; et al. New cytotoxic callipeltins from the Solomon Island marine sponge *Asteropus* sp. *Tetrahedron* **2016**, *72*, 6929-6934, doi:10.1016/j.tet.2016.09.016.
80. Clark, D.P.; Carroll, J.; Naylor, S.; Crews, P. An antifungal cyclodepsipeptide, Cyclolithistide A, from the sponge *Theonella swinhoei*. *J. Org. Chem.* **1998**, *63*, 8757-8764, doi:10.1021/jo980758p.
81. Urda, C.; Fernandez, R.; Rodriguez, J.; Perez, M.; Jimenez, C.; Cuevas, C. Daedophamide, a cytotoxic cyclodepsipeptide from a *Daedalopelta* sp. sponge collected in indonesia. *J. Nat. Prod.* **2017**, *80*, 3054-3059, doi:10.1021/acs.jnatprod.7b00678.
82. Zampella, A.; Sepe, V.; Luciano, P.; Bellotta, F.; Monti, M.C.; D'Auria, M.V.; Jepsen, T.; Petek, S.; Adeline, M.T.; Laprevote, O.; et al. Homophymine A, an anti-HIV cyclodepsipeptide from the sponge *Homophymia* sp. *J. Org. Chem.* **2008**, *73*, 5319-5327, doi:10.1021/jo800583b.
83. Zampella, A.; Sepe, V.; Bellotta, F.; Luciano, P.; D'Auria, M.V.; Cresteil, T.; Debitus, C.; Petek, S.; Poupat, C.; Ahond, A. Homophymines B-E and A1-E1, a family of bioactive cyclodepsipeptides from the sponge *Homophymia* sp. *Org. Biomol. Chem.* **2009**, *7*, 4037-4044, doi:10.1039/b910015f.
84. Rashid, M.A.; Gustafson, K.R.; Cartner, L.K.; Shigematsu, N.; Pannell, L.K.; Boyd, M.R. Microspinosamide, a new HIV-inhibitory cyclic depsipeptide from the marine sponge *Sidonops microspinosa*. *J. Nat. Prod.* **2001**, *64*, 117-121, doi:10.1021/np0002379.
85. Plaza, A.; Gustchina, E.; Baker, H.L.; Kelly, M.; Bewley, C.A. Mirabamides A-D, depsipeptides from the sponge *Siliquariaspongia* mirabilis that inhibit HIV-1 fusion. *J. Nat. Prod.* **2007**, *70*, 1753-1760, doi:10.1021/np070306k.
86. Lu, Z.; Van Wagoner, R.M.; Harper, M.K.; Baker, H.L.; Hooper, J.N.; Bewley, C.A.; Ireland, C.M. Mirabamides E-H, HIV-inhibitory depsipeptides from the sponge *Stelletta clavosa*. *J. Nat. Prod.* **2011**, *74*, 185-193, doi:10.1021/np100613p.
87. Okada, Y.; Matsunaga, S.; van Soest, R.W.; Fusetani, N. Nagahamide A, an antibacterial depsipeptide from the marine sponge *Theonella swinhoei*. *Org. Lett.* **2002**, *4*, 3039-3042, doi:10.1021/ol0262791.
88. Ford, P.W.; Gustafson, K.R.; McKee, T.C.; Shigematsu, N.; Maurizi, L.K.; Pannell, L.K.; Williams, D.E.; Dilip de Silva, E.; Lassota, P.; Allen, T.M.; et al. Papuamides A–D,

HIV-inhibitory and cytotoxic depsipeptides from the sponges *Theonella mirabilis* and *Theonella swinhoei* collected in papua new guinea. *J. Am. Chem. Soc.* **1999**, *121*, 5899-5909, doi:10.1021/ja990582o.

89. Prasad, P.; Aalbersberg, W.; Feussner, K.D.; Van Wagoner, R.M. Papuamides E and F, cytotoxic depsipeptides from the marine sponge *Melophlus* sp. *Tetrahedron* **2011**, *67*, 8529-8531, doi:10.1016/j.tet.2011.08.100.

90. Capon, R.J.; Ford, J.; Lacey, E.; Gill, J.H.; Heiland, K.; Friedel, T. Phoriospongin A and B: two new nematocidal depsipeptides from the Australian marine sponges *Phoriospongia* sp. and *Callyspongia bilamellata*. *J. Nat. Prod.* **2002**, *65*, 358-363, doi:10.1021/np010329d.

91. Coello, L.; Reyes, F.; Martin, M.J.; Cuevas, C.; Fernandez, R. Isolation and structures of pipecolidepsins A and B, cytotoxic cyclic depsipeptides from the madagascan sponge *Homophymia lamellosa*. *J. Nat. Prod.* **2014**, *77*, 298-303, doi:10.1021/np400888e.

92. Gulavita, N.K.; Gunasekera, S.P.; Pomponi, S.A.; Robinson, E.V. Polydiscamide A: a new bioactive depsipeptide from the marine sponge *Discodermia* sp. *J. Org. Chem.* **2002**, *57*, 1767-1772, doi:10.1021/jo00032a031.

93. Feng, Y.; Carroll, A.R.; Pass, D.M.; Archbold, J.K.; Avery, V.M.; Quinn, R.J. Polydiscamides B-D from a marine sponge *Ircinia* sp. as potent human sensory neuron-specific G protein coupled receptor agonists. *J. Nat. Prod.* **2008**, *71*, 8-11, doi:10.1021/np070094r.

94. Martin, M.J.; Rodriguez-Acebes, R.; Garcia-Ramos, Y.; Martinez, V.; Murcia, C.; Digon, I.; Marco, I.; Pelay-Gimeno, M.; Fernandez, R.; Reyes, F.; et al. Stellatolides, a new cyclodepsipeptide family from the sponge *Ecionemia acervus*: isolation, solid-phase total synthesis, and full structural assignment of stellatolide A. *J. Am. Chem. Soc.* **2014**, *136*, 6754-6762, doi:10.1021/ja502744a.

95. Nakamukai, S.; Takada, K.; Furihata, K.; Ise, Y.; Okada, S.; Morii, Y.; Yamawaki, N.; Takatani, T.; Arakawa, O.; Gustafson, K.R.; et al. Stellatolide H, a cytotoxic peptide lactone from a deep-sea sponge *Discodermia* sp. *Tetrahedron Lett.* **2018**, *59*, 2532-2536, doi:10.1016/j.tetlet.2018.05.033.

96. Shin, H.J.; Rashid, M.A.; Cartner, L.K.; Bokesch, H.R.; Wilson, J.A.; McMahon, J.B.; Gustafson, K.R. Stellettapeptins A and B, HIV-inhibitory cyclic depsipeptides from the marine sponge *Stelletta* sp. *Tetrahedron Lett.* **2015**, *56*, 4215-4219, doi:10.1016/j.tetlet.2015.05.058.

97. Ratnayake, A.S.; Bugni, T.S.; Feng, X.; Harper, M.K.; Skalicky, J.J.; Mohammed, K.A.; Andjelic, C.D.; Barrows, L.R.; Ireland, C.M. Theopapuamide, a cyclic depsipeptide from a Papua New Guinea lithistid sponge *Theonella swinhoei*. *J. Nat. Prod.* **2006**, *69*, 1582-1586, doi:10.1021/np060229d.

98. Suenaga, K.; Mutou, T.; Shibata, T.; Itoh, T.; Fujita, T.; Takada, N.; Hayamizu, K.; Takagi, M.; Irifune, T.; Kigoshi, H.; et al. Aurilide, a cytotoxic depsipeptide from the sea hare *Dolabella* auricularia: isolation, structure determination, synthesis, and biological activity. *Tetrahedron* **2004**, *60*, 8509-8527, doi:10.1016/j.tet.2004.06.125.

99. Sone, H.; Nemoto, T.; Ishiwata, H.; Ojika, M.; Yamada, K. Isolation, structure, and synthesis of dolastatin D ,a cytotoxic cyclic depsipeptide from the sea hare *dolabella abricularia Tetrahedron Lett.* **1993**, *34*, 8449-8452, doi:10.1016/s0040-4039(00)61356-4.

100. Sone, H.; Shibata, T.; Fujita, T.; Ojika, M.; Yamada, K. Dolastatin H and Isodolastatin H, potent cytotoxic peptides from the sea hare *Dolabella* auricularia: isolation,

stereostructures, and synthesis. *J. Am. Chem. Soc.* **1996**, *118*, 1874-1880, doi:10.1021/ja9519086.

101. Mutou, T.; Kondo, T.; Ojika, M.; Yamada, K. Isolation and stereostructures of dolastatin G and nordolastatin G, cytotoxic 35-membered cyclodepsipeptides from the Japanese sea hare *Dolabella auricularia*. *J. Org. Chem.* **1996**, *61*, 6340-6345, doi:10.1021/jo9608228.
102. Pettit, G.R.; Kamano, Y.; Herald, C.L.; Dufresne, C.; Bates, R.B.; Schmidt, J.M.; Cerny, R.L.; Kizu, H. Antineoplastic agents. 190. isolation and structure of the cyclodepsipeptide dolastatin 14. *J.Org.Chem* **2002**, *55*, 2989-2990, doi:10.1021/jo00297a001.
103. Hamann, M.T.; Otto, C.S.; Scheuer, P.J.; Dunbar, D.C. Kahalalides: bioactive peptides from a marine mollusk *Elysia* rufescens and its algal diet *Bryopsis* sp. *J. Org. Chem.* **1996**, *61*, 6594-6600, doi:10.1021/jo960877+.
104. Hamann, M.T.; Scheuer, P.J. Kahalalide F: a bioactive depsipeptide from the sacoglossan mollusk *Elysia rufescens* and the green alga *Bryopsis* sp. *J. Am. Chem. Soc.* **1993**, *115*, 5825-5826, doi:10.1021/ja00066a061.
105. Horgen, F.D.; delos Santos, D.B.; Goetz, G.; Sakamoto, B.; Kan, Y.; Nagai, H.; Scheuer, P.J. A new depsipeptide from the sacoglossan mollusk *Elysia ornata* and the green alga *Bryopsis* species. *J. Nat. Prod.* **2000**, *63*, 152-154, doi:10.1021/np990402o.
106. Tilvi, S.; Naik, C.G. Tandem mass spectrometry of kahalalides: identification of two new cyclic depsipeptides, kahalalide R and S from *Elysia* grandifolia. *J. Mass. Spectrom.* **2007**, *42*, 70-80, doi:10.1002/jms.1140.
107. Rao, K.V.; Na, M.K.; Cook, J.C.; Peng, J.; Matsumoto, R.; Hamann, M.T. Kahalalides V-Y isolated from a Hawaiian collection of the sacoglossan mollusk *Elysia* rufescens. *J. Nat. Prod.* **2008**, *71*, 772-778, doi:10.1021/np070508g.
108. Ciavatta, M.L.; Devi, P.; Carbone, M.; Mathieu, V.; Kiss, R.; Casapullo, A.; Gavagnin, M. Kahalalide F analogues from the mucous secretion of Indian sacoglossan mollusc *Elysia ornata*. *Tetrahedron* **2016**, *72*, 625-631, doi:10.1016/j.tet.2015.12.003.
109. Kimura, J.; Takada, Y.; Inayoshi, T.; Nakao, Y.; Goetz, G.; Yoshida, W.Y.; Scheuer, P.J. Kulokekahilide-1, a cytotoxic depsipeptide from the cephalaspidean mollusk *Philinopsis speciosa*. *J. Org. Chem.* **2002**, *67*, 1760-1767, doi:10.1021/jo010176z.
110. Nakao, Y.; Yoshida, W.Y.; Takada, Y.; Kimura, J.; Yang, L.; Mooberry, S.L.; Scheuer, P.J. Kulokekahilide-2, a cytotoxic depsipeptide from a cephalaspidean mollusk *Philinopsis* speciosa. *J. Nat. Prod.* **2004**, *67*, 1332-1340, doi:10.1021/np049949f.
111. Nakao, Y.; Yoshida, W.Y.; Szabo, C.M.; Baker, B.J.; Scheuer, P.J. More peptides and other diverse constituents of the marine mollusk *Philinopsis* speciosa. *J. Org. Chem.* **1998**, *63*, 3272-3280, doi:10.1021/jo9719867.
112. Rodríguez, J.; Fernández, R.; Quiñoá, E.; Riguera, R.; Debitus, C.; Bouchet, P. Onchidin: a cytotoxic depsipeptide with C2 symmetry from a marine mollusc. *Tetrahedron Lett.* **1994**, *35*, 9239-9242, doi:10.1016/0040-4039(94)88477-3.
113. Fernández, R.; Rodríguez, J.; Quiñoá, E.; Riguera, R.; Muñoz, L.; Fernández-Suárez, M.; Debitus, C. Onchidin B: a new cyclodepsipeptide from the mollusc *Onchidium*

sp. *J. Am. Chem. Soc.* **1996**, *118*, 11635-11643, doi:10.1021/ja961314i.

114. Nakao, Y.; Yoshida, W.Y.; Scheuer, P.J. Pupukeamide, a linear tetrapeptide from a cephalaspidean mollusk *Philinopsis* speciosa. *Tetrahedron Lett.* **1996**, *37*, 8993-8996, doi:10.1016/s0040-4039(96)02111-9.
115. Kim, M.Y.; Sohn, J.H.; Ahn, J.S.; Oh, H. Alternaramide, a cyclic depsipeptide from the marine-derived fungus *Alternaria* sp. SF-5016. *J. Nat. Prod.* **2009**, *72*, 2065-2068, doi:10.1021/np900464p.
116. Lin, Y.; Wang, J.; Wu, X.; Zhou, S.; Vrijmoed, L.L.P.; Jones, E.B.G. A novel compound Enniatin G from the mangrove fungus *Halosarpheia* sp. (strain #732) from the south China sea. *Aust. J.Chem.* **2002**, *55*, 225-227, doi:10.1071/ch01164.
117. Zhu, X.; Zhong, Y.; Xie, Z.; Wu, M.; Hu, Z.; Ding, W.; Li, C. Fusarihexins A and B: novel cyclic hexadepsipeptides from the mangrove endophytic fungus *Fusarium* sp. R5 with antifungal activities. *Planta. Med.* **2018**, *84*, 1355-1362, doi:10.1055/a-0647-7048.
118. Amagata, T.; Morinaka, B.I.; Amagata, A.; Tenney, K.; Valeriote, F.A.; Lobkovsky, E.; Clardy, J.; Crews, P. A chemical study of cyclic depsipeptides produced by a sponge-derived fungus. *J. Nat. Prod.* **2006**, *69*, 1560-1565, doi:10.1021/np060178k.
119. Feng, Y.; Blunt, J.W.; Cole, A.L.; Munro, M.H. A novel cyclodepsipeptide, HA23, from a *Fusarium* sp. *Org. Lett.* **2002**, *4*, 2095-2096, doi:10.1021/ol0260167.
120. Kawahara, T.; Takagi, M.; Shin-Ya, K. Three new depsipeptides, JBIR-113, JBIR-114 and JBIR-115, isolated from a marine sponge-derived *Penicillium* sp. fS36. *J. Antibiot.* **2012**, *65*, 147-150, doi:10.1038/ja.2011.126.
121. Belofsky, G.N.; Jensen, P.R.; Fenical, W. Sansalvamide: A new cytotoxic cyclic depsipeptide produced by a marine fungus of the genus *Fusarium*. *Tetrahedron Lett.* **1999**, *40*, 2913-2916, doi:10.1016/s0040-4039(99)00393-7.
122. Nihei, K.; Itoh, H.; Hashimoto, K.; Miyairi, K.; Okuno, T. Antifungal cyclodepsipeptides, W493 A and B, from *Fusarium* sp.: isolation and structural determination. *Biosci. Biotechnol. Biochem.* **1998**, *62*, 858-863, doi:10.1271/bbb.62.858.
123. Lv, F.; Daletos, G.; Lin, W.; Proksch, P. Two new cyclic depsipeptides from the endophytic fungus *Fusarium* sp. *Nat. Prod. Commun.* **2015**, *10*, 1667-1670.
124. Oh, D.-C.; Jensen, P.R.; Fenical, W. Zygosporamide, a cytotoxic cyclic depsipeptide from the marine-derived fungus *Zygosporium masonii*. *Tetrahedron Lett.* **2006**, *47*, 8625-8628, doi:10.1016/j.tetlet.2006.08.113.
125. Zhou, Z.; Wang, X.; Zhang, H.; Sun, J.; Zheng, L.; Liu, H.; Wang, J.; Shen, A.; Geng, M.; Guo, Y. Chromopeptide A, a highly cytotoxic depsipeptide from the marine sediment-derived bacterium *Chromobacterium* sp. HS-13-94. *Acta. Pharm Sin B* **2015**, *5*, 62-66, doi:10.1016/j.apsb.2014.11.001.
126. Hu, J.-F.; Wunderlich, D.; Sattler, I.; Feng, X.-Z.; Grabley, S.; Thiericke, R. Rakicidin C, A new cyclic depsipeptide from *Streptomyces* sp. *Eur. J. Org. Chem* **2000**, *2000*, 3353-3356, doi:10.1002/1099-0690(200010)2000:19<3353::Aid-ejoc3353>3.0.Co;2-e.
127. Igarashi, Y.; Shimasaki, R.; Miyanaga, S.; Oku, N.; Onaka, H.; Sakurai, H.; Saiki, I.; Kitani, S.; Nihira, T.; Wimonsiravude, W.; et al. Rakicidin D, an inhibitor of tumor cell

invasion from marine-derived *Streptomyces* sp. *J. Antibiot.* **2010**, *63*, 563-565, doi:10.1038/ja.2010.70.

128. Kitani, S.; Ueguchi, T.; Igarashi, Y.; Leetanasaksakul, K.; Thamchaipenet, A.; Nihira, T. Rakicidin F, a new antibacterial cyclic depsipeptide from a marine sponge-derived *Streptomyces* sp. *J. Antibiot.* **2017**, 1-3, doi:10.1038/ja.2017.92.
129. Chen, L.; Zhao, W.; Jiang, H.-L.; Zhou, J.; Chen, X.-M.; Lian, Y.-Y.; Jiang, H.; Lin, F. Rakicidins G - I, cyclic depsipeptides from marine *Micromonospora chalcea* FIM 02-523. *Tetrahedron* **2018**, *74*, 4151-4154, doi:10.1016/j.tet.2018.06.039.
130. Trischman, J.A.; Tapiolas, D.M.; Jensen, P.R.; Dwight, R.; Fenical, W.; McKee, T.C.; Ireland, C.M.; Stout, T.J.; Clardy, J. Salinamides A and B: anti-inflammatory depsipeptides from a marine *streptomycete*. *J. Am. Chem. Soc.* **2002**, *116*, 757-758, doi:10.1021/ja00081a042.
131. Hassan, H.M.; Degen, D.; Jang, K.H.; Ebright, R.H.; Fenical, W. Salinamide F, new depsipeptide antibiotic and inhibitor of bacterial RNA polymerase from a marine-derived *Streptomyces* sp. *J. Antibiot.* **2015**, *68*, 206-209, doi:10.1038/ja.2014.122.
132. Machida, K.; Arai, D.; Katsumata, R.; Otsuka, S.; Yamashita, J.K.; Ye, T.; Tang, S.; Fusetani, N.; Nakao, Y. Sameuramide A, a new cyclic depsipeptide isolated from an ascidian of the family Didemnidae. *Bioorgan. Med. Chem.* **2018**, *26*, 3852-3857, doi:10.1016/j.bmc.2018.06.042.
133. Kodani, S.; Komaki, H.; Hemmi, H.; Miyake, Y.; Kaweewan, I.; Dohra, H. Streptopeptolin, a cyanopeptolin-type peptide from *Streptomyces* olivochromogenes. *ACS. Omega.* **2018**, *3*, 8104-8110, doi:10.1021/acsomega.8b01042.
134. Tiwari, A.; Kaweewan, I.; Miyake, Y.; Hemmi, H.; Kodani, S. Isolation and structure determination of new chymotrypsin inhibitory peptides streptopeptolins B and C. *Nat. Prod. Res.* **2021**, *35*, 2700-2706, doi:10.1080/14786419.2019.1663514.
135. Romero, F.; Espliego, F.; Perez Baz, J.; Garcia de Quesada, T.; Gravalos, D.; de la Calle, F.; Fernandez-Puentes, J.L. Thiocoraline, a new depsipeptide with antitumor activity produced by a marine *Micromonospora*. I. Taxonomy, fermentation, isolation, and biological activities. *J. Antibiot.* **1997**, *50*, 734-737, doi:10.7164/antibiotics.50.734.
136. Wyche, T.P.; Hou, Y.; Braun, D.; Cohen, H.C.; Xiong, M.P.; Bugni, T.S. First natural analogs of the cytotoxic thiodepsipeptide thiocoraline A from a marine *Verrucosispora* sp. *J. Org. Chem.* **2011**, *76*, 6542-6547, doi:10.1021/jo200661n.
137. Oku, N.; Kawabata, K.; Adachi, K.; Katsuta, A.; Shizuri, Y. Unnarmicins A and C, new antibacterial depsipeptides produced by marine bacterium *Photobacterium* sp. MBIC06485. *J. Antibiot.* **2008**, *61*, 11-17, doi:10.1038/ja.2008.103.
138. Nair, V.; Kim, M.C.; Golen, J.A.; Rheingold, A.L.; Castro, G.A.; Jensen, P.R.; Fenical, W. Verrucosamide, a cytotoxic 1,4-thiazepane-containing thiodepsipeptide from a marine-derived *Actinomycete*. *Mar. Drugs* **2020**, *18*, 549, doi:10.3390/md18110549.
139. Kan, Y.; Fujita, T.; Sakamoto, B.; Hokama, Y.; Nagai, H. Kahalalide K: A new cyclic depsipeptide from the hawaiian green alga *bryopsis* species. *J. Nat. Prod.* **1999**, *62*, 1169-1172, doi:10.1021/np990053y.

140. Dmitrenok, A.; Iwashita, T.; Nakajima, T.; Sakamoto, B.; Namikoshi, M.; Nagai, H. New cyclic depsipeptides from the green alga *Bryopsis* species; application of a carboxypeptidase hydrolysis reaction to the structure determination. *Tetrahedron* **2006**, *62*, 1301-1308, doi:10.1016/j.tet.2005.10.079.
141. Iwasaki, A.; Ohno, O.; Sumimoto, S.; Matsubara, T.; Shimada, S.; Sato, T.; Suenaga, K. Mebamamides A and B, cyclic lipopeptides isolated from the Green Alga *Derbesia* marina. *J. Nat. Prod.* **2015**, *78*, 901-908, doi:10.1021/acs.jnatprod.5b00168.
142. Li, Y.; Yu, H.B.; Zhang, Y.; Leao, T.; Glukhov, E.; Pierce, M.L.; Zhang, C.; Kim, H.; Mao, H.H.; Fang, F.; et al. Pagoamide A, a cyclic depsipeptide Isolated from a cultured marine chlorophyte, *Derbesia* sp., using MS/MS-based molecular networking. *J. Nat. Prod.* **2020**, *83*, 617-625, doi:10.1021/acs.jnatprod.9b01019.
